# Investigating and Modeling the Factors That Affect Genetic Circuit Performance

**DOI:** 10.1021/acssynbio.3c00151

**Published:** 2023-11-02

**Authors:** Shai Zilberzwige-Tal, Pedro Fontanarrosa, Darya Bychenko, Yuval Dorfan, Ehud Gazit, Chris J. Myers

**Affiliations:** † The Shmunis School of Biomedicine and Cancer Research, Life Sciences Faculty, Tel Aviv University, Tel Aviv-Yafo 6997801, Israel; ‡ Department of Electrical, Computer, and Energy Engineering, University of Colorado Boulder, Boulder, Colorado 80309, United States; § Bio-engineering, Electrical Engineering Faculty, Holon Institute of Technology (HIT), Holon 5810201, Israel; ∥ Alagene Ltd., Innovation Center, Reichman University, Herzliya 7670608, Israel

**Keywords:** genetic circuit, DBTL, outside-the-lab, robustness, redesign, model predictions

## Abstract

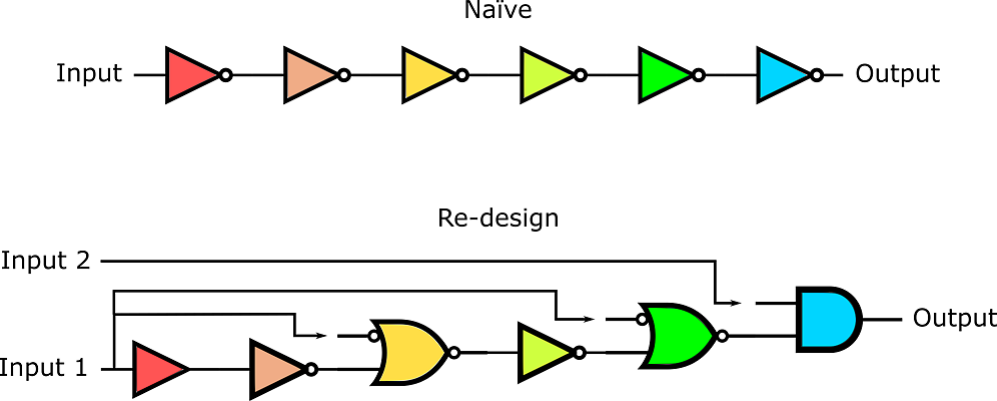

Over the past 2 decades, synthetic biology has yielded ever more complex genetic circuits that are able to perform sophisticated functions in response to specific signals. Yet, genetic circuits are not immediately transferable to an outside-the-lab setting where their performance is highly compromised. We propose introducing a broader test step to the design–build–test–learn workflow to include factors that might contribute to unexpected genetic circuit performance. As a proof of concept, we have designed and evaluated a genetic circuit in various temperatures, inducer concentrations, nonsterilized soil exposure, and bacterial growth stages. We determined that the circuit’s performance is dramatically altered when these factors differ from the optimal lab conditions. We observed significant changes in the time for signal detection as well as signal intensity when the genetic circuit was tested under nonoptimal lab conditions. As a learning effort, we then proceeded to generate model predictions in untested conditions, which is currently lacking in synthetic biology application design. Furthermore, broader test and learn steps uncovered a negative correlation between the time it takes for a gate to turn ON and the bacterial growth phases. As the synthetic biology discipline transitions from proof-of-concept genetic programs to appropriate and safe application implementations, more emphasis on test and learn steps (i.e., characterizing parts and circuits for a broad range of conditions) will provide missing insights on genetic circuit behavior outside the lab.

## Introduction

Synthetic biology aims to address pressing global challenges including disease diagnosis and treatment,^1,2^ biofuel production,^3−5^ contamination detection,^6^ and biomanufacturing.^4,7−11^ These are achieved by engineering biological systems with new capabilities, granting cellular control and user-defined performance.^4,12^ The variety of genetic circuit functions include a genetic toggle switch,^13^ genetic counters,^14^ low- or high-frequency filters,^15,16^ adders,^17^ sequential asynchronous logic circuits,^18^ and more.

An implicit, iterative *Design–Build–Test–Learn* (DBTL) process is often used to develop these ingenious genetic circuits.^19−22^ However, bias is introduced into the DBTL process in almost all of its steps, and the variability of environmental factors that affect a circuit’s behavior is often not considered. For example, fluorescence proteins are used as reporter genes. Fluorescence protein signals can be affected by several factors such as pH, salt concentration, and the presence of other molecules.^23−27^ Therefore, it is crucial to take into account the behavior of the reporter protein under different conditions when designing a circuit. This may impede the circuit’s expected performance when applied in outside-the-lab (OTL) settings under conditions that mimic real-world environments. In addition, models used by genetic design automation (GDA) tools are mostly based on characterization experiments carried out under very specific and restricted optimal lab conditions (OLCs).^28−31^ These restricted characterizations of genetic parts can lead to a misleading design process aimed at OTL applications, which include untested conditions. Thus, in turn, unexpected behaviors can occur.^19,32^ In non-OLCs, the behavior of the designed circuit can produce erroneous or faulty performance with unpredictable outcomes. Furthermore, with a narrow *Test* step suited for OLC, the *learning* usually is limited to a post hoc description of circuit dynamics. This would be especially perilous for engineered systems that are aimed at operating in dynamic environments, such as living therapeutics and whole-cell biosensors. Broader test experimental conditions, emulating different context ranges that the designed circuit might be expected to encounter, would help in producing more accurate model parameters to obtain better part and system-wide predictions for both tested and untested experimental conditions. Moreover, the range of experimental conditions can aid researchers in identifying patterns of trends related to specific metabolic mechanisms and processes. This, in turn, can help designers focus their efforts to countermeasure these effects in the design, to avoid circuit failures or malfunctions.^33^

Aiming to address the unexpected behavior of genetic circuits when applied in nonoptimal conditions, this study applies a broader *Test* step to a designed delay-signal circuit. The *Test* step includes more environmental dynamic factors, a genetic circuit that might encounter OTL conditions, and two different reporting systems. The circuit’s output, as well as the time for output detection, was observed to be highly variable for different temperatures, media, inducer concentration, bacterial growth phases, and output reporters. If the performance of the delay circuit is compromised by the tested experimental factors presented here, it will inevitably alter its behavior in other contexts, which would not have been predicted by GDA tools. Most studies either have a nonexistent *Learn* step, or it consists only of a post hoc description of the designed circuit performance at OLCs.

This work not only provides a reparametrization effort for different experimental conditions but also generates a new model able to predict the outcome of untested conditions. We demonstrate how this process is extremely manual and labor-intensive and argue that a more systematic and automated workflow should be discussed and developed for synthetic biology workflows. As a case study, we focused on the effect of bacterial growth phases on the circuit behavior. We observed that the different growth phases affected the total output production as well as the output detection time. This, in turn, allowed for a deeper *Learn* step, which uncovered a negative correlation between part production rates and growth phases. Using these parameter trends enabled the prediction of the circuit output production for untested experimental conditions. Additionally, the discovered trend and new model may indicate an unknown underlying biological mechanism affecting the rate and total production of the circuit output, which could be further studied.

Thus, we propose that greater emphasis on the *Test* and *Learn* steps of a DBTL workflow is needed to build more predictive models and to reduce bias across the entire DBTL workflow enabling the possibility of finding design alternatives to unexpected behavior and performance when the circuits are used in applications, improving a genetic circuit robustness.^19^ As we move from proof-of-concept designs to more real-life applications, a thorough *Test* step provides the necessary data that allow for a significant *Learn* step.

## Results

### Designed Circuit and Predicted Behavior

**Figure 1:**
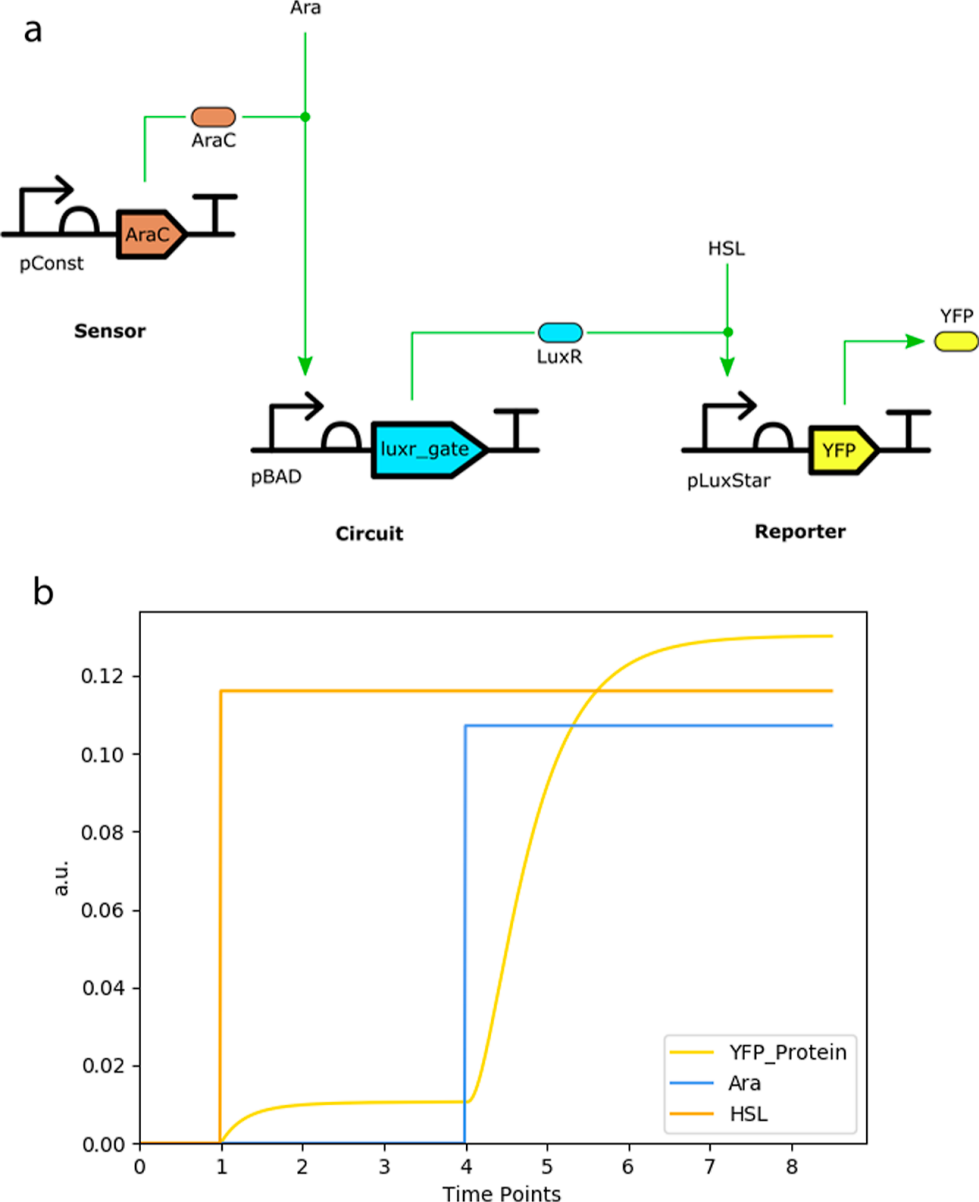
(a) Designed delay circuit using Cello gates.^29^ Sequences obtained from SynBioHub.^34^ This circuit produces YFP after a delay when both Ara and HSL are present. (b) Delay circuit simulation results were obtained using default parameters.^a^ YFP production (in au) over increasing simulated time points (arbitrary to iBioSim).

Figure 1a shows a schematic of the actual circuit designed, built, and tested in this work. For more information on the layout design, please refer to the Methods section.

The intended purpose of this design is to provide some delay between an input concentration change and output production while avoiding unwanted switching behavior due to the initial propagation of an erroneous state. According to this design, the circuit does not produce *yellow fluorescent protein* (YFP), unless both *arabinose* (Ara) and *oxohexanoyl-homoserine lactone* (HSL) are present. This design avoids unwanted production of output (YFP), when the circuit is initialized in a cell without Ara, even if there is initial production of LuxR. If there is no HSL, the circuit will not produce YFP. Both Ara and HSL are needed to produce the circuit output.

The initial model predictions of the circuit, shown in Figure 1b, were done in iBioSim^35^ using an automatic model generator to produce an *ordinary differential equation* (ODE) model of the circuit. The resulting complete model was then analyzed using the Runge–Kutta–Fehlberg method,^36^ also implemented in iBioSim.^35^

The simulation results show that there is no YFP production when only HSL is present, and furthermore, there is a delay in the YFP production when Ara is added as expected. However, given that these simulations are using default parameters that were characterized under OLCs (obtained from ref (29)), these provide qualitative information on how the actual circuit is going to behave only when tested in OLCs.

### Control Experiment

**Figure 2:**
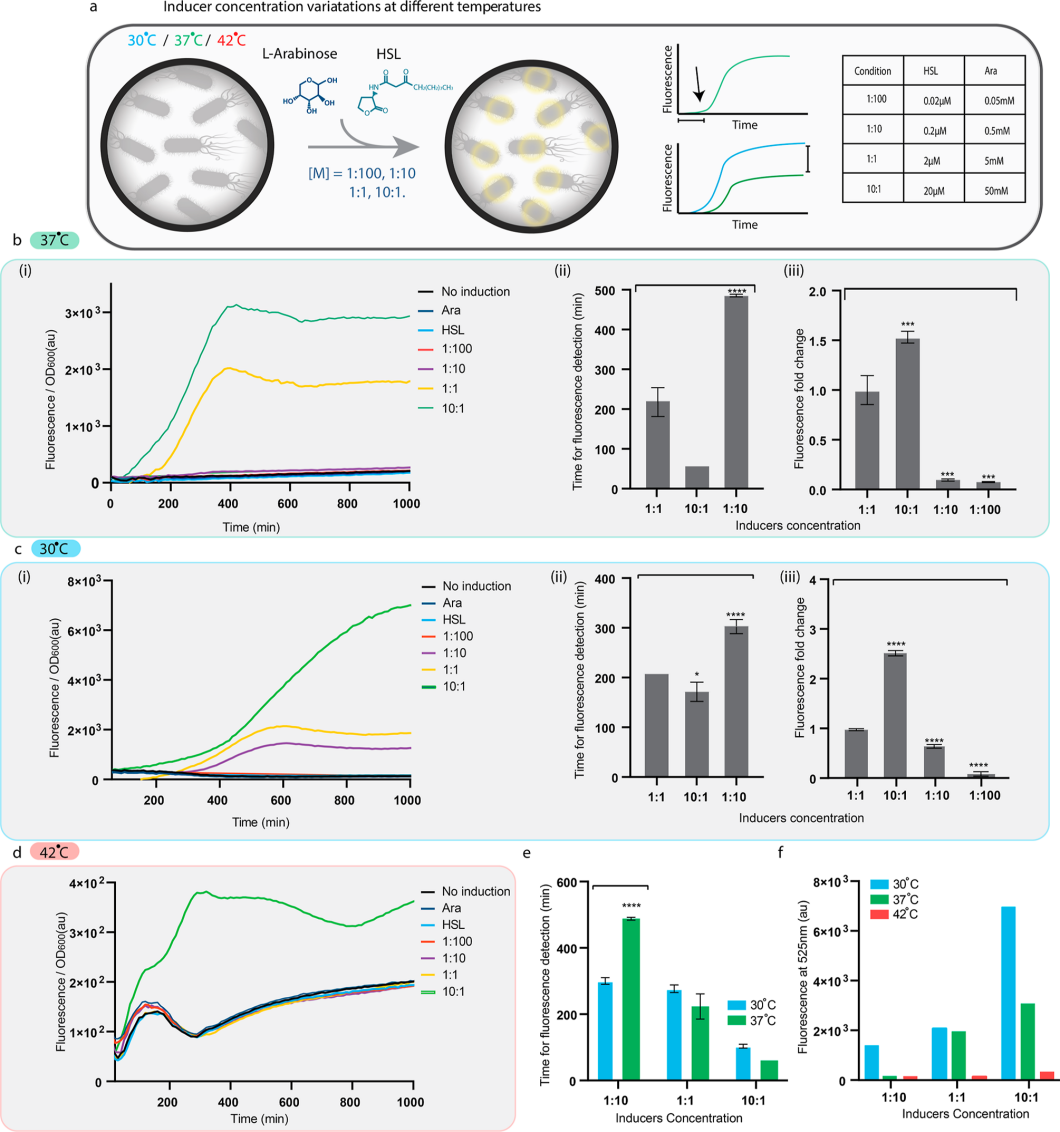
Control assay and inducer concentration variations at different temperatures. (a) Scheme of the control and inducer concentration variation assays, the measured parameters, and inducer concentrations. Time for fluorescence detection was determined as the time it took to detect a fluorescence signal above the threshold of the negative control. The fluorescence fold change of the maximum fluorescence signal of the different samples was compared to the maximum fluorescence signal of the optimal condition sample. Ranging inducer concentrations (as specified in the table) were added at *T* = 0, 37, 30, and 42 °C. (b) Measured fluorescence signal (in au) over time (i) of the control assay and of bacteria that were cultivated with a range of inducer concentrations. (b) (ii) Comparison of the time to detect a fluorescence signal from bacteria cultivated with different inducer concentrations. *P < 0.01, ***P < 0.005 (Student’s *t*-test). (b) (iii) Comparison of the maximum fluorescence signal intensity fold change detected from bacteria that were cultivated with different inducers concentrations. ***P < 0.005 (Student’s *t*-test). (c) (i) Measured fluorescence signal (in au) over time of bacteria cultivated with different inducer concentrations at 30 °C. (c) (ii) Comparison of the time to detect a fluorescence signal. *P < 0.01. ***P < 0.005 (Student’s *t*-test). (c) (iii) Comparison of the maximum fluorescence signal intensity. ***P < 0.005, ****P < 0.001 (Student’s *t*-test). (d) Measured fluorescence signal (in au) over time of bacteria cultivated with different inducer concentrations at 42 °C. The differences between the negative controls and the induced samples were not significant and therefore cannot be plotted in fluorescence detection and fold change graphs. (e) Comparison of the time to detect a fluorescence signal at 30 and 37 °C from bacteria cultivated with different inducer concentrations. (f) Comparison of maximum fluorescence signal at 30, 37, and 42 °C from bacteria cultivated with different inducer concentrations.

Using iBioSim^35^ and standard genetic parts,^29^ the delay circuit was designed aiming for a relative output time delay postinduction. A simple control experiment characterizing genetic parts was set using the OLC to test the actual delay. Briefly, bacteria were cultivated in M9 glucose media at 37 °C in the presence of both inducers from *T* = 0 (Ara and HSL), which simulate the characterization assays that were done in Cello^29^ (Figure 2a). As negative controls, the bacteria were also cultivated without any inducer or in the presence of only one of the inducers. The fluorescence was normalized by subtracting the average blank value from the average fluorescence value and dividing the resulting fluorescence value by the average OD600 value (which represents the number of bacteria) for each time point. The normalized fluorescence values of the samples from *T* = 0 onward were then compared to the normalized fluorescence values of the negative control (without induction). Under the conditions we tested as optimal, it took an average of 180 min to detect a fluorescence signal [as shown in Figure 2b(i),(ii)]. We have set 180 min as the *optimal detection time* (ODT) and the maximum signal intensity from this assay as the *optimal intensity* (OI), since both were measured under optimal growth conditions.

Next, the circuit robustness was tested under different conditions that mimic the dynamic environments in which bacteria may encounter the OTL. The following sections describe the genetic circuit dynamic performance under different conditions. The purpose of this work is to examine how these conditions affect the detection time and the signal intensity.

### Inducer Concentrations

An important dynamic condition that bacteria could encounter when applying the OTL is the inducer concentration. The inducer concentrations that were set in our control experiment as 1:1 are 5 mM and 2 μM for Ara and HSL, respectively, since these are the concentrations that were used in Cello.^29^ The bacteria were cultivated in the presence of serially diluted concentrations from 10× the standard concentration to 1:100 (10:1, 1:1, 1:10, and 1:100) (Figure 2a). Bacteria that were cultivated in the presence of the 10:1 inducers were able to produce a fluorescence signal much faster than the ODT [Figure 2b(i),(ii)]. In addition, the signal intensity of the 10:1 sample was significantly higher than the signal from the OI [Figure 2b(iii)]. However, in the lower concentrations of 1:100 and 1:10, the fluorescence signal was weak and barely detected using our methods [Figure 2b(i),(iii)]. Thus, the genetic circuit behavior in terms of time for signal detection and its intensity is highly compromised and dependent on the inducer concentrations. Thus, the genetic circuit behavior is not robust, in terms of time for signal detection and signal intensity.

### Temperature

Among environmental conditions, the temperature is a crucial variable affecting microbial growth.^37^ When temperatures exceed optimal levels, the heat shock response triggers the temporary production of proteins that protect cells from damage and reduce protein synthesis as the cells allocate resources to safeguard themselves and rectify any destruction caused by the high temperature. When the temperature decreases, the cold shock response is activated. The proteins produced during this response increase both the rate of gene transcription and protein production, helping cells adapt to the lower temperatures.^38−41^ Based on the effects temperature has on bacterial growth as well as gene expression patterns, we hypothesize that temperature will also affect the genetic circuit behavior. *Escherichia coli* can survive in a range of temperatures starting from 4 °C up to 45 °C, with an optimal growth at 37 °C, and it is usually the temperature used for cultivating *E. coli* in the lab.^42^ To test our hypothesis, *E. coli* was cultivated at 30 and 42 °C in the presence of a range of inducer concentrations as described above (Figure 2a). Lowering the growth temperature from 37 to 30 °C resulted in a higher fluorescence signal of both 10:1 and 1:10 inducer concentrations (Figure 2c). The circuit’s behavior was then tested at 42 °C in the presence of different inducer concentrations as mentioned above. At this temperature, a fluorescence signal was observed only in the high inducer concentration (10:1) (Figure 2d). It should be noted that in addition to a lower fluorescence signal of the 1:10 dilution at 37 °C, the time to detect the signal was significantly higher (Figure 2e), and the intensity of the detected signal at 42 °C was an order of magnitude lower than the resulting OI (Figure 2f). We assume that the burden of the genetic circuit on the bacteria is higher at 42 °C than 37 °C, since in the higher temperature the bacteria is under greater stress due to increased expression of heat shock proteins, which add to the overall burden on the cell.^43^ Moreover, there is a positive correlation between the peptide elongation rate and the temperature, as the rate increases with temperature up to 37 °C. However, beyond 37 °C, the rate remains constant.^37^ This indicates that at high temperatures, the rate of peptide chain elongation does not limit growth, suggesting that the reduction in protein synthesis at high temperatures is not due to a restriction in the rate of protein synthesis. Rather, it is a result of other factors, such as high protein degradation rates or other metabolic changes occurring in response to high temperatures.

### Soil

**Figure 3:**
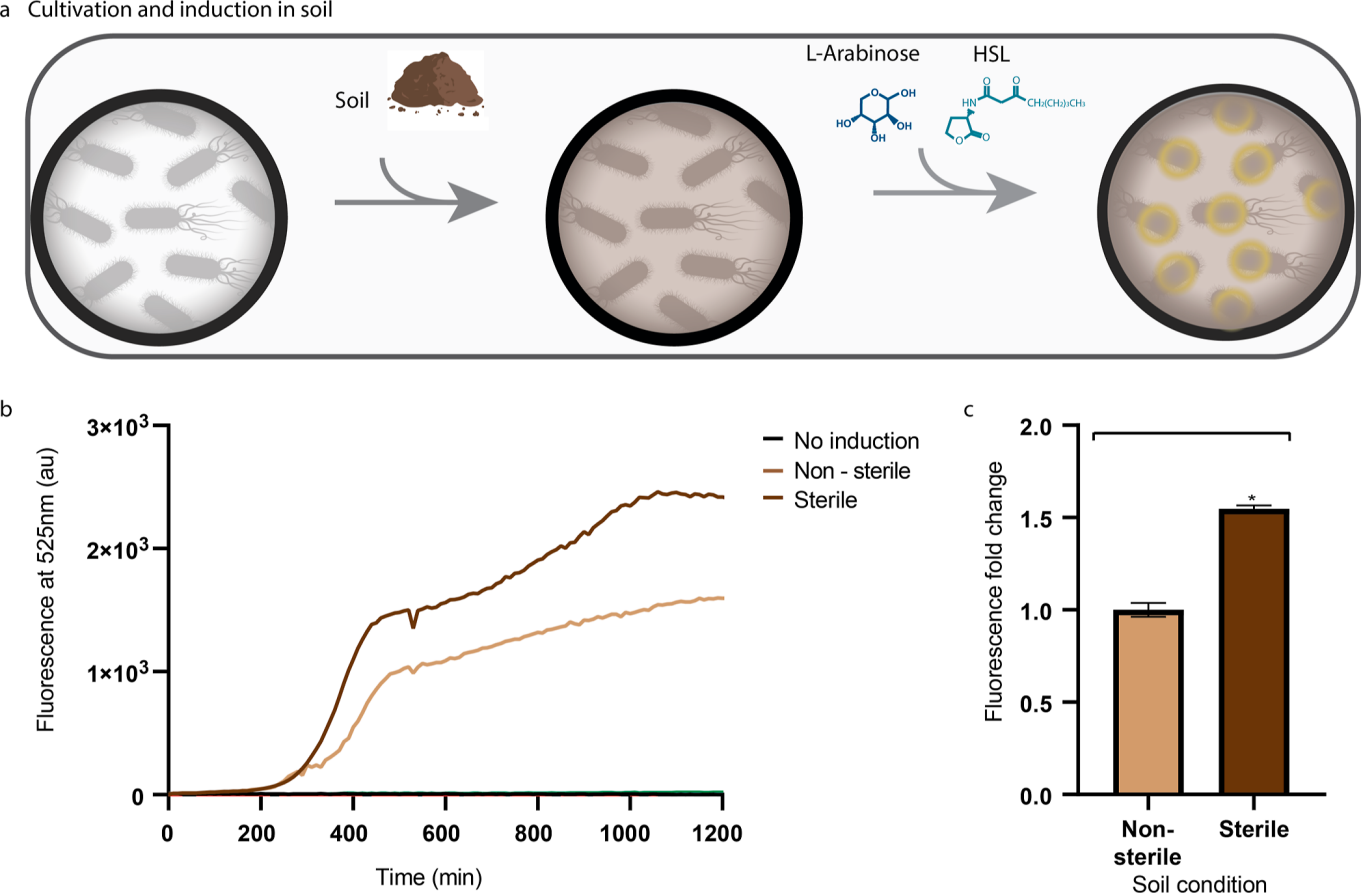
Cultivation in media containing 2% sterile and nonsterile soil. (a) Scheme of the cultivation with soil assay, 2% (W/V) soil was added to media and then both HSL and Ara were added at *T* = 0, which induced the production of YFP. (b) Measured fluorescence signal (in au) over time of the bacteria cultivated in 2% (W/V) sterile and nonsterile soil. (c) Comparison of the maximum fluorescence signal intensity detected from bacteria that were cultivated in 2% sterile and nonsterile soil. *P < 0.01 (Student’s *t*-test).

Medium composition can be highly diversified, which may lead to an altered genetic circuit behavior. We decided to test our genetic circuit in a medium that will mimic applications of the OTL, such as soil. *E. coli* was cultivated in both sterile and nonsterile soil (Figure 3a) testing the effect of other bacteria in the soil samples. A fluorescence signal was detected in both samples; however, the signal intensity was higher in the sterile soil sample (Figure 3b,c). This is probably because in the sterile sample there is less competition for nutrients since the sample was autoclaved prior to the addition of the *E. coli*. While in the nonsterile sample, there are many other microorganisms competing for nutrients, which lead to a lower signal.

### Induction at Different Bacterial Growth Phases

**Figure 4:**
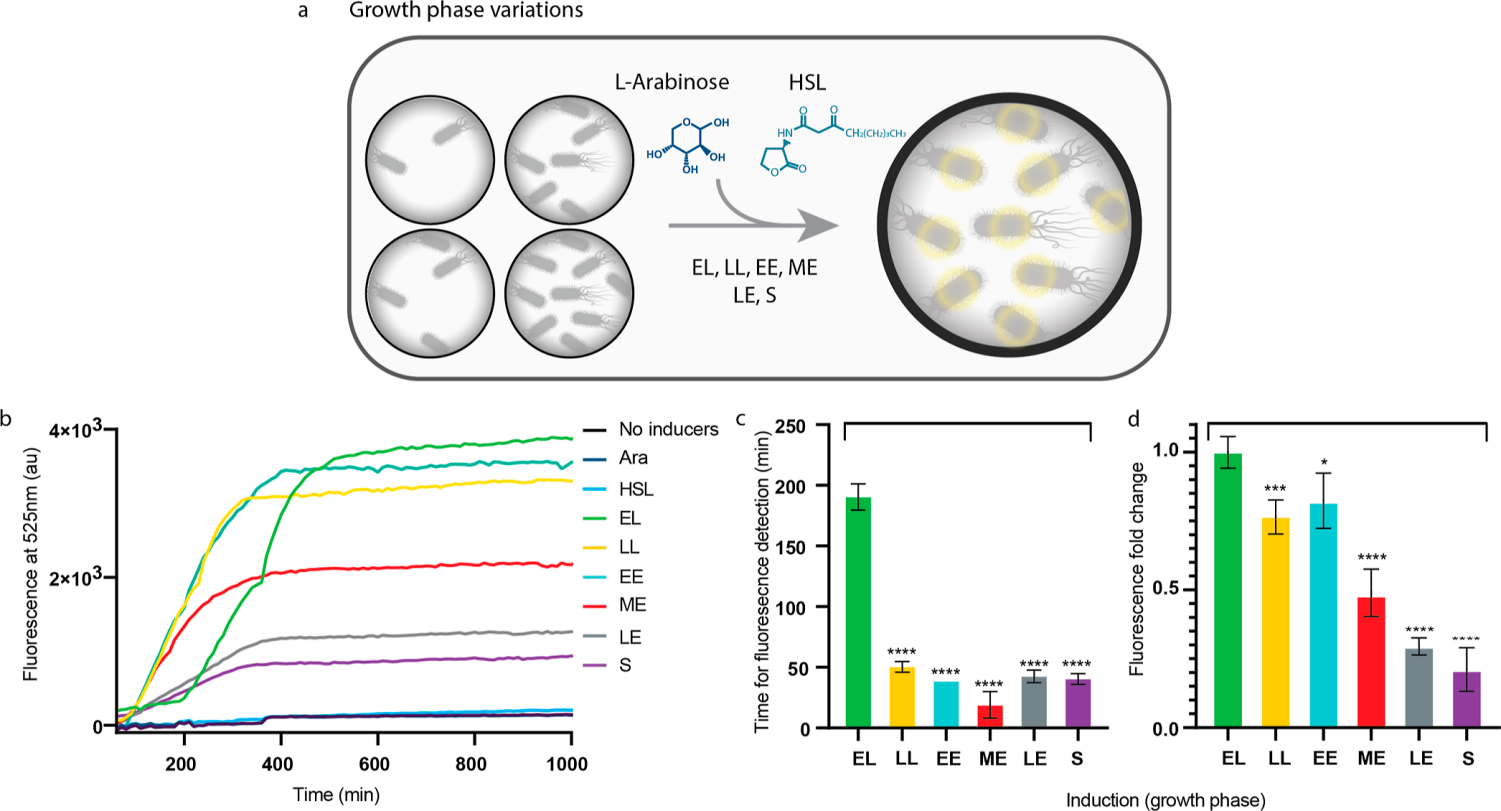
Growth phase variation assay. (a) Scheme of the growth phase variations. HSL was added at *T* = 0 (EL) and Ara was later on added at a different growth phase which induced the production of YFP. (b) Measured fluorescence signal (in au) over time of bacteria induced at different growth phases. (c) Comparison of the time to detect a fluorescence signal from the bacteria. ****P < 0.001 (Student’s *t*-test). (d) Comparison of the maximum fluorescence signal intensity fold change detected from the bacteria. *P < 0.01, ***P < 0.005, ****P < 0.001 (Student’s *t*-test).

Optical density (OD) measurements are usually used for quantifying the growth of a bacterial culture.^44^ When the data are plotted semilogarithmic, four growth phases are distinguishable: (i) the lag phase which is nonreplicative; (ii) the exponential phase which is replicative; (iii) the stationary phase where growth ceases, but cells remain metabolically active, and (iv) the death phase where there is a gradual decline in viable cells. During these phases, the transcriptome and proteome of the bacteria dramatically change as well as the transcription and translation rates. To test the robustness of the time delay circuit across different growth phases, we grew the bacteria in the presence of HSL from *T* = 0, and we added the second inducer, Ara, at different growth phases: early lag (*T* = 0, such as the control experiment), late-lag, early exponential, midexponential, late-exponential, and stationary and measured the fluorescence signal over time (Figure 4a). When the inducers were added at a later phase than the early lag (*EL*), which is also the ODT, the time for fluorescence detection was significantly decreased (Figures 4b and Figure S2). Interestingly, we observed that the decrease was gradual from late-lag (*LL*) to early exponential (EE) and middle-exponential (ME). This was followed by a minor increase from late-exponential (LE) and stationary (*S*) (Figure 4c). When the signal intensities of the different induction times are compared, it is clearly shown that the signal decreases when induction starts at a later stage, especially at ME, LE, and *S* (Figure 4b,d). The induction at various growth stages did not have an impact on the doubling time of the bacteria when compared to the doubling time without induction (Figure S1). The behavior of the time-delay genetic circuit alters when the bacteria are induced at different growth phases, suggesting that the unsteadiness of the bacteria transcription and translation rates influences the behavior of the genetic circuit.

### Total Fluorescence Production

**Figure 5:**
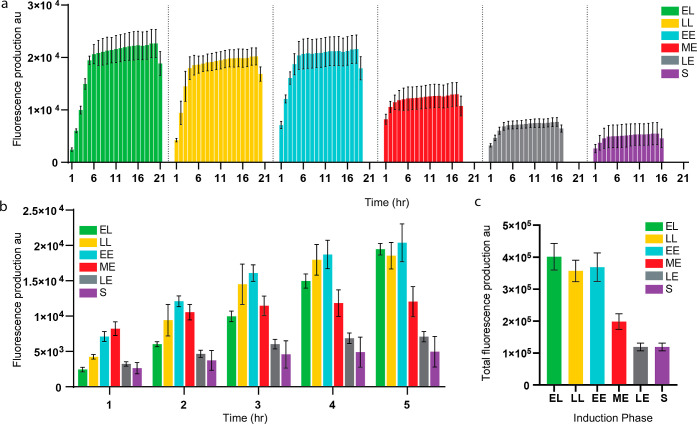
Accumulating fluorescence (au) production of bacteria induced at different growth phases. (a) Hourly accumulated fluorescence (au) of bacteria induced at different growth phases. (b) Comparison of the hourly accumulated fluorescence (au) of the first 5 h. (c) Comparison of the total accumulated fluorescence (au). ****P < 0.001 (Student’s *t*-test).

To address the different genetic circuit behavior when induced at different growth phases, we calculated the total amount of fluorescence in *arbitrary units* (au) that are produced hourly following the detection of the fluorescence signal (see Methods). Our hypothesis is that genetic circuits that are induced during the exponential phase, in which bacteria are dividing rapidly and thus producing more proteins, the hourly production rate will be higher than genetic circuits that are induced in the lag or stationary phase, where protein synthesis is much lower.^44^ Moreover, genetic circuits that are induced at a later growth phase will not produce as much total fluorescence as genetic circuits that are induced earlier since the bacteria are closer to the stationary phase and start to die due to the lack of nutrients. Thus, we decided to compare the accumulated fluorescence production in bacteria that are induced at different growth phases (Figure 5a). According to the results, in the first few hours, the highest fluorescence production was of LL and EE induction (Figure 5b). However, following 4 h, the hourly fluorescence production of EL, LL, and EE was relatively similar (Figure 5b). Through the first 5 h, the LE and S hourly fluorescence production were the lowest (Figure 5b). These findings are consistent with our hypothesis regarding the susceptibility of the bacteria to efficiently produce a signal if the induction occurs in an early growth phase. Moreover, the sum of the hourly productions further emphasizes that bacteria that are induced at a later stage (starting from the ME phase) are more likely to produce less signal overall (Figure 5c). Thus, the range of the circuit behavior is heavily influenced by the specific growth phase in which the bacteria encounter the signal molecules. We hypothesize that the growth phase status is an immensely important factor with implications for protein production and degradation rates, which directly affect gate production rates. This means that the growth phase status will affect the parameter values used in the model and therefore the predicted behavior. We decided to characterize the gate production rates at different growth phases (see below).

### Gate Reparametrization

The effects of various growth phases on the production delays and signal intensities of the circuit were studied in this work. To achieve this, we performed characterization experiments for each growth phase. These experiments were performed following the methods described in Shin et al.^45^ (see Methods). As a result of these experiments, we obtained new parameter values corresponding to each growth phase, which are detailed below.

### Hill Function Parameters

The mathematical models for the internal gates are taken from Shin et al.,^45^ which describe the input and output fluxes (RPU)^29,46^ of each gate using a Hill function-based equation.^47,48^eq 1 describes the steady-state output of a genetic gate as
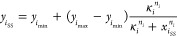
1where *y*_*i*_SS__ is the steady-state output RNAP flux of gate *i*; *y*_*i*_min__ and *y*_*i*_max__ are the minimal and maximal output RNAP fluxes, respectively, for gate *i*; κ_*i*_ and *n*_*i*_ are obtained from the affinity and cooperative of transcription factor binding; and, finally, *x*_*i*SS_ is the input RNAP flux from the input promoter, which in the case of this work, is also calculated using eq 1. eqs 2 and 3 are variations of eq 1, which have been used to calculate the steady-state output of a genetic gate.^29,45,48^ To acquire the Hill function parameter values used in this equation, gate induction experiments were performed and then fitted as shown in the “Parametrization Methods” section. The estimation of Hill function parameter values for different gates was obtained by fitting eq 2 (for *repression*) and 3 (for *activation*) to part-characterization experiments using plasmids shown in Figure S6 at different growth phases (EL and LE) as explained in the Results section.
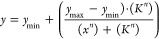
2
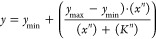
3

The Hill function fit parameter values obtained are shown in Table S3. These parameters can be used to calculate the *steady-state* output of the circuit. The Hill function parameter values obtained using this method differ in only 1 order of magnitude for some cases (for *n* and κ) and 2 orders of magnitude (for *y*_min_ and *y*_max_) from the ones published in Shin et al.,^45^ which is expected due to the different measurement instrumentation used and are system/laboratory specific. The next section, however, will describe the model used to predict the *dynamical* behavior of genetic circuits (i.e., predicted behavior *between* steady states).

### Tau (τ) Parameters

To describe how quickly each gate reaches the steady-state output, the parameters τ_ON_ and τ_OFF_ can be used to describe how quickly a gate turns ON or OFF, respectively.^45^ The dynamic parameter values for different gates were estimated by fitting eqs 4 and 5 to ON-to-OFF and *OFF-to-ON* part-characterization experiments at different bacterial growth phases (EL, LL, EE, ME, LE, and S) as explained in the Results section of this work. The experiments used the gate plasmids shown in Figure S6. These characterizations were performed to determine if the dynamic behavior of the designed circuits varies with growth phases. The reported parameter values are rounded to three significant digits. These experiments can be used to fit the data using eqs 4 and 5, obtained from the Supporting Information in Shin et al.,^45^ to achieve parameter values for these conditions. The results obtained are shown in Table S4.
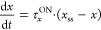
4
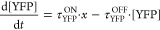
5

**Figure 6:**
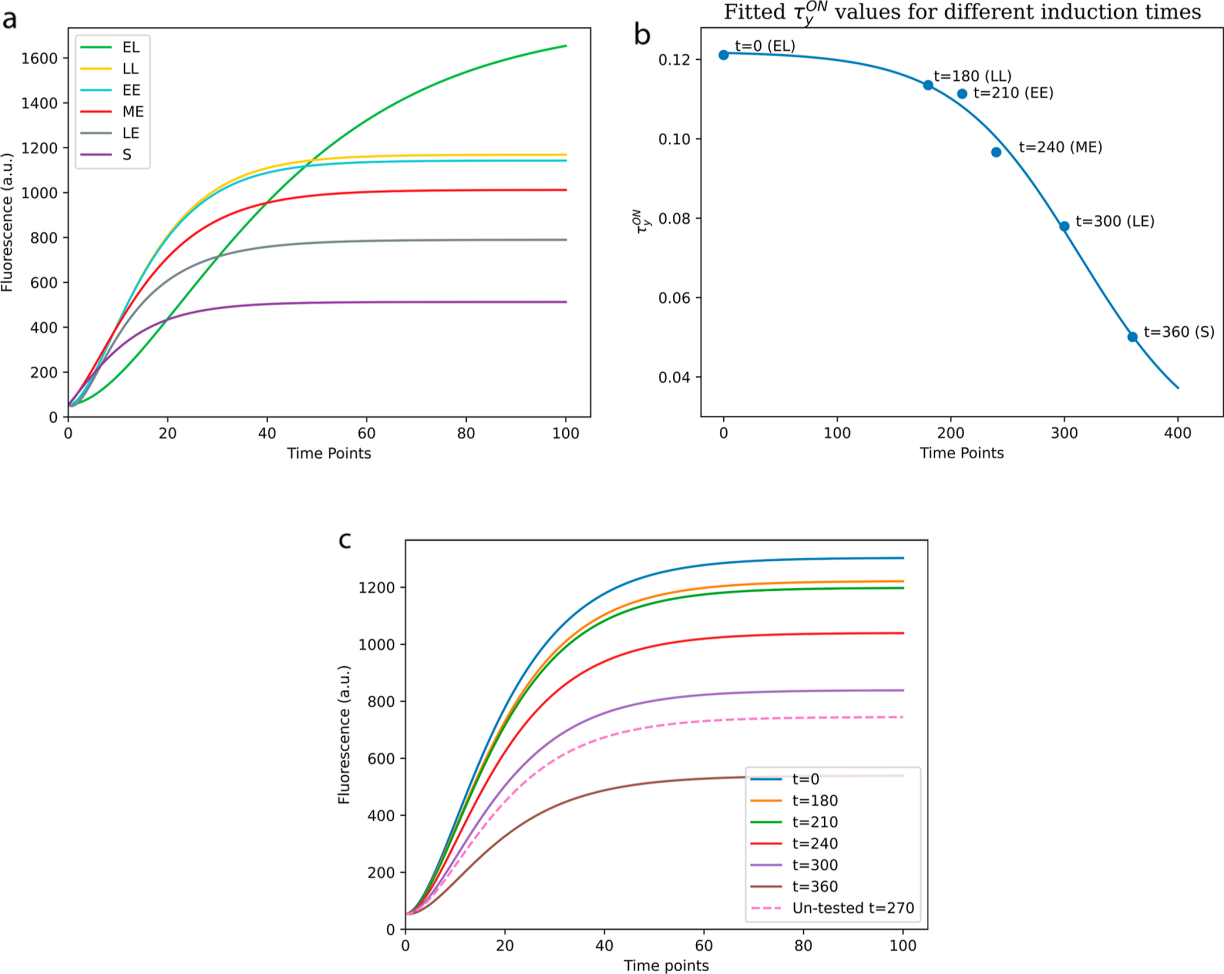
(a) Simulation results for the different growth phases of the AraC gate, using fitted parameters shown in Table S3, obtained using the lmfit Python package.^49^ (b) Fitted τ_*y*_^ON^ values obtained when fitting fluorescence values for different induction times (*t* = 0, *t* = 180, *t* = 210, etc.) using fitted parameters obtained using the lmfit Python package.^49^ (c) Untested YFP output prediction for induction time of 270 min. This untested prediction was obtained using the fitted (and fixed) parameter values (Figure S5) and the model shown in the Results section, except for the τ_*y*_^ON^ value which is unknown for *t* = 270. To estimate the τ_*y*_^ON^ value for the untested condition (*t* = 270), the sigmoid model of eq 6 with the fitted parameter values was used.

Using the parameters shown in Table S3, derived from the fitting algorithm, new simulations were produced for each growth phase (Figure 6a). The new model simulations predict both lower production of YFP protein (signal intensity) and the decrease in time for reaching the steady state for each successive growth phase as observed experimentally.

However, the parametrization results (shown in Table S3) were produced without fixing any of the parameter values when using the fitting algorithm, meaning that they were all treated as free variables. When there are no fixed parameters, then the fitting algorithm will find the best fit by manipulating the parameter values until the expected outcome best matches the experimental results. This will result in widely different parameter values to compensate for other parameters’ minimization of error while fitting (see, for example, *x*_SS_ values for different growth phases in Table S3). This means it will be hard to derive any parameter value trends indicative of what might be happening to their magnitude as the circuit is induced at different growth phases.

To discern whether there are any parameter value trends that can be attained from fitting the experimental results, we proceeded to fix parameter values to reduce the number of free variables in the fitting algorithm. The first parameter value fixed was τ_*y*_^OFF^. This was done using the fitting algorithm with the ON-to-OFF gate characterization experiments (see the Methods section). Refitting the model was refit to the experimental results, with τ_*y*_^OFF^ as a fixed parameter value and the rest as free variables, new parameter values were acquired. These refitting iterations were done then by fixing subsequent parameter values by calculating averages from previous iterations. First, *x*_SS_ average values were calculated, fixed for the next iteration, followed by τ_*x*_^ON^. With the last iteration of this refitting process, τ_*y*_^ON^ was left as a free variable, while the rest of the parameters were fixed. This process was carried out to understand the effect of the different experimental conditions on the value of τ_*y*_^ON^. First, since τ_*y*_^ON^ is the parameter closest to the measured parameters in the experiments (YFP fluorescence); and second, because if the other parameter values are not fixed, then if there are any parameter value trends, it is lost in the minimization process, when the fitting algorithm tries to find the solutions by increasing a parameter value and decreasing another one. Figure 6b shows the τ_*y*_^ON^ parameter values obtained following this procedure. We selected the order for subsequent parameter value fixations until only τ_*y*_^ON^ was left as a free parameter, through trial and error. This was done until we observed a trend in the free parameters. The fitted parameter values for each of these iterations are shown in Table S5.

As hypothesized, the values of τ_*y*_^ON^ shown in Figure 6b vary for the different growth phases of the clonal bacteria. If all parameters are unrestricted, then there is significant variation in all of them under different experimental conditions. Nonetheless, we found that modifying τ_*y*_^ON^ could account for most of the observed changes. Therefore, changes in the τ_*y*_^ON^ value can largely explain the experimental variations. When bacteria are induced at a later growth phase, the values of τ_*y*_^ON^ decrease. This coincides with previously observed experimental results, where induction at later growth phases decreases the time it took to reach the steady state, as well as the maximum signal intensity at the steady state. A predictive sigmoid model of signal intensity over time can be created as shown in eq 6
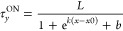
6where τ_*y*_^ON^ is the predicted value of gate dynamics when the circuit evolves to an *ON* state^45^ and *x* is the independent variable *inductiontime*. Using the Scipy Python package^50^ to fit eq 6 to the fitted values of τ_*y*_^ON^ for the different growth phases (as shown in Figure 6b), *L* was estimated to be 1.02423886 × 10^–1^, *x*_0_ equal to 3.13060089 × 10^2^, *k* equal to 1.80082383 × 10^–2^, and *b* to be 1.95602872 × 10^–2^. With this equation, a researcher could estimate the value of τ_*y*_^ON^ for different times of induction and therefore estimate the decrease in output production and delay of a delay circuit for untested conditions as shown in Figure 6c.

These model predictions suggest that the different growth phases affect the time scale by which a gate turns on (τ^ON^ parameter values), which could be correlated to the increased transcription of genes of the lag and exponential phases of cell growth, and the decreased transcription rates of the stationary phase.^51^ This, in turn, affects the speed at which the circuit produces the intended output as well as the total amount of output production (as seen in Figure 6a).

### Lysis

**Figure 7:**
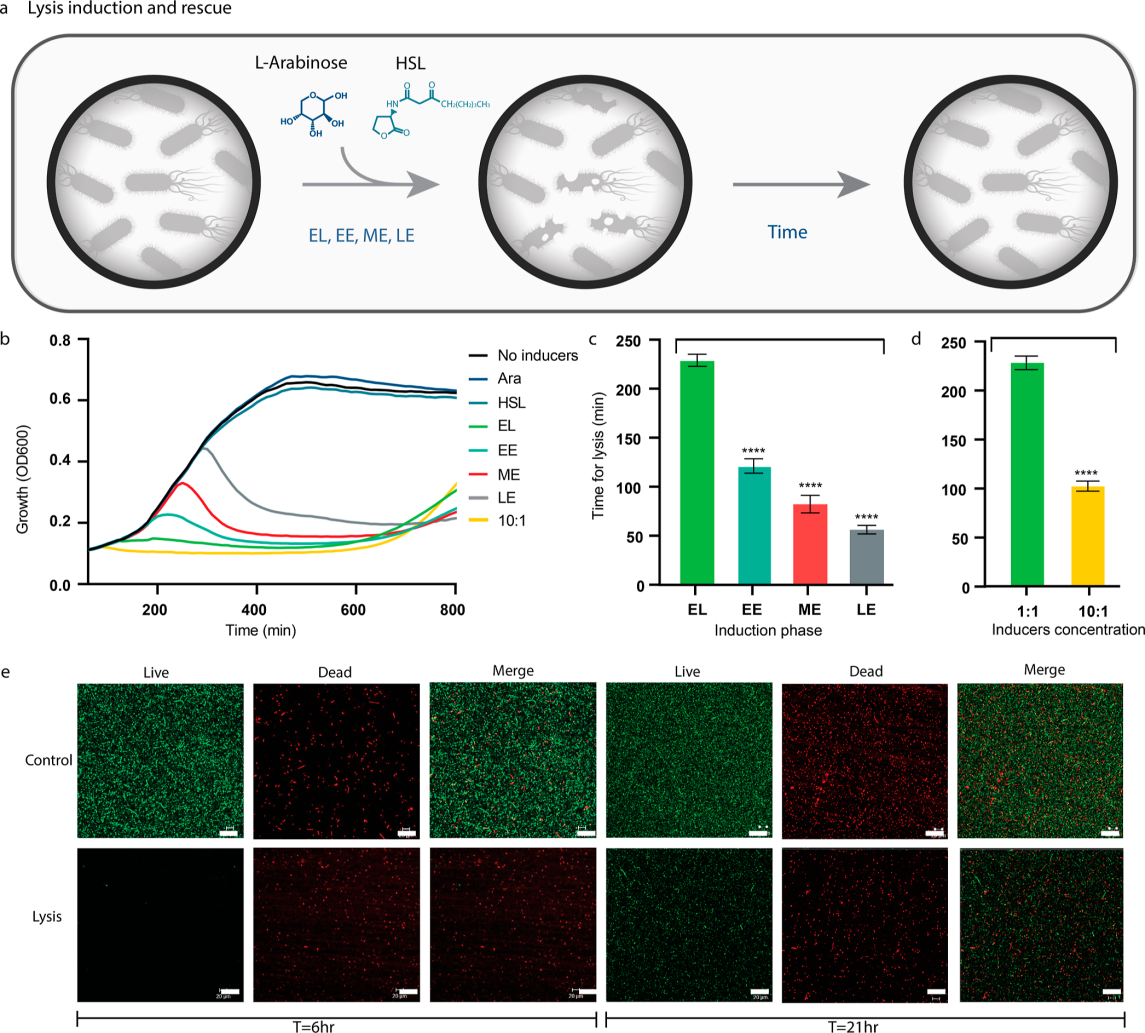
Lysis induction was performed under different conditions. (a) Scheme of the lysis assay. HSL was added at *T* = 0, Ara was added later at different growth phases, which induced lysis. With time, some bacteria overcame the lysis process, which is shown with OD. Addition of Ara; *T* = 0; EL and 1:10, *T* = 180; EE, *T* = 210; ME, *T* = 240; LE. (b) Bacteria growth over time of the different bacteria induced at various growth phases. (c,d) Comparison of the time it took to achieve a decrease in the OD of the bacteria at different growth phases (c) and inducer concentrations (d). ****P < 0.001 (Student’s *t*-test). (e) Confocal images of a live–dead assay of control bacteria (without induction) and lysed bacteria at two different time points. The scale bar is 40 μm.

The significance of accurately timing and delaying the expression of lysis proteins by engineered bacteria as a biocontainment safety mechanism in the field of synthetic biology is well acknowledged.^52^ However, an understanding of the specific factors that affect the timing and precision of this process is limited. Therefore, we tested the circuit behavior when producing a lysis protein rather than YFP. We designed a circuit that produces the MS2 lysis protein L (see Figure S3). Although MS2 bacteriophage is one of the most researched bacteriophages, the mechanisms underlying protein L lysis abilities remain mainly unknown.^53^ Similar to the YFP assay setup, HSL was present in media from *T* = 0 and Ara was added at different growth phases and at different concentrations, and the time for lysis was measured (see Methods) (Figure 7a,b). When the inducers were added at the EL phase, the time for lysis was 240 min; however, when the inducers were added at later growth phases, there was a gradual significant decrease in the time it took to detect lysis (Figure 7c). Thus, the induction at later growth phases alters the circuit behavior and leads to faster lysis. Similarly, when a 10:1 concentration was added, the bacteria were lysed faster than the bacteria cultivated with a 1:1 concentration (Figure 7d).

The OD was measured for 800 min, and around 650 min there was an increase in the OD across all samples, which led us to believe that the bacteria escaped the lysis process, probably due to mutation insertions in the circuit plasmids. It should be noted, that the time it took the bacteria to rescue itself from lysis was the highest for EL induction (*T* = 0) and the 10:1 inducer concentrations (also at *T* = 0) (Figure S4). This can be explained by the initial small amount of bacteria at *T* = 0. Samples from different time points were taken to confocal microscopy for a live–dead assay and compared to a noninduced sample (Figure 7e). The results show indeed that following 6 h from induction, the vast majority of bacteria were dead. However, following 21 h, there was an increase in the amount of live bacteria, further validating the ability of the bacteria to escape lysis (Figure 7b,e). These results indicate that for efficient biocontainment control, the L protein of the MS2 bacteriophage should be combined with an additional killing mechanism, regardless of the different induction conditions.

## Discussion

This paper argues that designs tested under an OLC may not work for OTL conditions. Additionally, there is a lack of a standardized and/or systematized workflow to understand how these conditions can affect genetic circuit behavior. We illustrate this point by describing our experience designing a delay circuit, which performed as expected in an OLC, but showed varying behavior in OTL conditions. While the authors understand that the current work does not present a fully fledged systematic approach for designing gene circuits suitable for environments outside the laboratory, we hope it serves as an earnest entreaty to the synthetic biology community to emphasize the “Learn” phase of the DBTL cycle, particularly if the objective is to progress toward engineering gene circuits for real-world applications in variable environments. Through the characterization of constituent parts and elucidation of their interactions, this work demonstrates the feasibility of predicting the behavior of synthetic gene circuits under varied conditions, albeit in a preliminary fashion. Furthermore, the intent of the paper is to lay the groundwork by emphasizing the criticality of testing and learning modalities in synthetic biology and the development of predictive models. These underpinnings are essential in ensuring that the field advances with informed strategies. The authors also convey that there is an ongoing effort to establish a more comprehensive, systematic, and automatic approach for the design and analysis of gene circuits beyond controlled laboratory conditions. This nascent approach, which is in development, aims to integrate rigorous testing and learning methodologies, quantitative modeling, and iterative design paradigms. It is envisioned that this framework will serve as a crucial step in translating the fundamental principles of synthetic biology into pragmatic applications in diverse environments.

To understand how different experimental conditions can affect the circuit behavior, we suggest that a broader test phase is necessary. We propose characterizing parts under different lab conditions to better understand how they might behave in the OTL settings. However, we acknowledge that this process is manual and labor-intensive, which can be a barrier for researchers without expertise in machine learning and PSA analysis. Thus, we call for the development of an automatic and systematic approach to this problem, including an automated machine learning process/workflow to allow a more systematic approach to the learning step in the DBTL workflow in synthetic biology. Furthermore, an automated or facilitated Test–Learn workflow will allow machine learning workflows to discover unexpected relationships between different laboratory conditions and genetic parts’ behavior that would otherwise be very hard to manually test for each possible combination. Ultimately, we argue that a more comprehensive understanding of circuit behavior under varying conditions will facilitate the development of synthetic biology systems for OTL use.

The DBTL workflow is a powerful methodology commonly and successfully implemented in the synthetic biology field. However, it is not complete and requires constant re-examination and updating to minimize the turnaround of synthetic biology applications. As an example, we engineer bacteria to act as a biosensor for a specific molecule and try to understand the effects of changing different laboratory conditions on the delay and signal intensity of the circuit. The time for fluorescence detection and signal intensity varies greatly for different experimental conditions, and it takes a lot of meticulous fitting and model generation to try to understand some of the phenomenological processes occurring. This work, for example, shows that if a bacteria was sensing a molecule at the EE stage and was observed prior to its ability to produce a fluorescence signal, then it could lead to a false negative result. In addition, bacteria that sensed the molecule at the *S* phase and therefore produced a significantly lower fluorescence signal could also lead to a false negative result. Furthermore, our results support the notion that when designing a genetic circuit, the range of inducer concentrations that can lead to a satisfying performance can vary across different cultivation temperatures, which is a major factor when transitioning to an OTL. Additionally, in this work, we hypothesized that the values of τ_*y*_^ON^ shown in Figure 6a vary for the different growth phases of the clonal bacteria. After learning from the appropriate test results, we could acquire new models in the *Learn* step that could predict the behavior of these circuits under untested experimental conditions. This alone can help determine the general robustness of a designed genetic circuit for OTL settings. However, comparing the predicted τ_*y*_^ON^ parameter values for the different growth phases also showed a decreasing trend that could point to the underlying mechanisms that affect the rate and total production of the circuit output. This could help designers find ways to rectify this effect if the intended purpose of the circuit needs to meet some critical values.

By expanding the scope of the Test step to encompass a wider range of environmental factors, we can enhance the overall learning process and gain a more comprehensive understanding of the challenges that genetic circuit performance encounters when it is transitioned to OTL conditions. Expansion of the Test step will inevitably promote new prediction models and tools developed for the scaling of genetic circuit production, which will be able to predict the behavior of genetic circuits under different conditions (even untested ones) and therefore will enable better design choices than those currently provided by GDA tools. Moreover, a systematized *Test* step for synthetic biology can generate the required data for automated machine learning processes of the *Learn* step, which would greatly facilitate the endeavor of understanding the effects of these conditions on circuit performance. Currently, there is a lack of standardized methodologies and/or software tools to help researchers perform a meaningful production scaling, and there is no consensus on what these methodologies should look like nor tools to help with this process. Even popular GDA tools (like Cello^29,54,55^), which provide extensive and automated *Design*, *Build*, and even *Test* steps, lack of a proper *Learn* step, which would be beneficial for researchers to come up with better design choices for OTL genetic circuit applications.

We propose the development of an automated system that can test the impact of environmental factors on the behavior of genetic devices. This system can run standardized experiments, including the variation of temperature, medium, inducer concentrations, growth phases of bacteria, and any other experimental conditions that initial tests reveal. The system could be implemented using laboratory automation or a software platform such as a software toolkit. The results from these experiments can be analyzed to determine the impact of each environmental factor on the behavior of the genetic device. Additionally, the system could also be designed to run a series of experiments that vary multiple factors simultaneously, allowing researchers to determine the combined effect of the parameters on the behavior of the genetic device. Machine learning approaches can also be used to understand which conditions have an effect on genetic parts. Although the current study did not address this issue, we acknowledge its importance and will consider incorporating it into future work. We believe that such a system would provide a useful tool for researchers to quickly and easily determine the impact of environmental factors on the behavior of genetic devices, and it could be adapted for use with different circuits and realizations.

We envision that these automated Learn step processes could be an integral part of future GDA tools for synthetic biology, which will accommodate the specific concerns. By standardizing and systematizing the Test step and automating the Learn step, researchers can more efficiently and effectively design genetic circuits that perform reliably under OTL conditions. This will allow for the development of more robust and versatile synthetic biology systems that can be used in a wider range of real-world applications. With the continued advancement of synthetic biology, such tools and workflows will become increasingly important for unlocking the full potential of this rapidly evolving field.

## Methods

### Circuit Design

**Figure 8:**
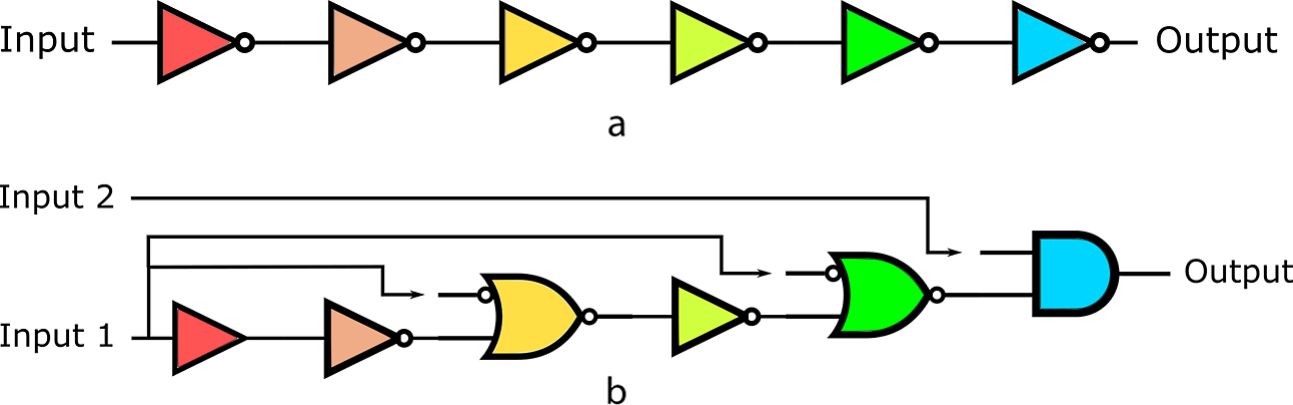
(a) Simple delay circuit. Two successive NOT gates (represented as) add delay to a circuit without changing the circuit’s behavior. In this image, each logic gate is represented with a different color to represent different gate assignments. (b) Setup of a failure-free delay circuit. This figure represents a NOT gate, an NIMPLY gate, and AND gate, and a *buffer* gate. Each logic gate is represented with a different color to represent different gate assignments.

A naive implementation of delay in a genetic circuit is shown in Figure 8a, where successive pairs of NOT gates can be used to add delay to a circuit (from a change of inputs to a change in outputs) without changing the circuits’ function or behavior. However, this circuit can produce unwanted output production (*setup* glitches) when it is initialized, even in the absence of input molecules, since its components have not been stabilized yet.^33^ This means that when the circuit is initialized since the circuit is not stabilized, some internal gates will start randomly producing output before others. This can cause an erroneous or faulty initial state for the circuit and therefore an unexpected or unwanted output protein production. So, for example, the gate that produces the output protein of a circuit (blue gate, Figure 8a) could start producing output before the circuit reaches the steady state without any inducer (input) present, at which point it will be repressed by the green gate.

However, it is possible to redesign the circuit in a way to avoid these initialization problems and properly lock the initial state down, so that there is no unwanted switching behavior, or setup glitches, when this circuit is initialized. Figure 8b shows such a design that would avoid setup failures due to the initialization problem. When such a circuit is transformed into a bacteria and there is random production from internal gates since the circuit is not in a steady state, there will be no unwanted output production. This is because the output-producing gate is an AND gate, which needs the presence of two signals before it can produce an output signal. The second inducer is necessary so that even if there is some initial leakage production of the green gate, the output is not going to be produced. The Results section shows the implementation of Figure 8b using Cello gates.^29^

### Mathematical Model

The model used by the automatic model generator of this work is based on a combination of a steady-state model developed in Nielsen et al.^29^ and Shin et al.,^45^ with a dynamic model developed by Moser et al.^56^ The modeling and simulation in this work use Cello genetic parts and parametrization, but it can work for any genetic circuit as long as the appropriate parameters are available.

The mathematical model used in this work is explained and implemented in Fontanarrosa et al.^33^ However, certain adaptations have been made to this model to be able to account for a “split” sensor gate in the design of the delay circuit. The model developed in Shin et al.^45^ represents sensor and internal genetic gates differently: while internal gates are modeled from a Hill-function-like equation, sensor gates are modeled as either being *ON* or *OFF*. Therefore, we adapted the model in order to have a Hill-function-like equation for sensor gates too in order to model the circuit as shown in Figure 8b. This circuit’s second-to-last gate (green gate), is a sensor gate, which was not initially intended to be an internal gate.^29^

The model uses response functions to describe the steady-state RNAP flux output (in RPUs) of a gate over the *output promoter* (promoter that the gate has an effect on), as a function of the RNAP fluxes of the input promoters (for greater detail, see ref (33)). However, for the model developed in Shin et al.,^45^ the steady-state calculation of input (sensor) promoter activities of input gates takes the following form
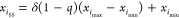
7where *x*_*i*_SS__ is the steady-state output RNAP flux of sensor gate *i*; *x*_*i*_min__ and *x*_*i*_max__ are the minimal and maximal output RNAP fluxes, respectively, for sensor gate *i*; *q* is the presence (*q* = 1) or absence (*q* = 0) of inducer molecules; and δ(1 – *q*) is 1 when there are inducer molecules present and 0 when there are not. This formula shows that the response function for a sensor gate is *digital*: it is either *ON* or *OFF*, and there is no response curve. This formulation would not work if a sensor gate is used as an *internal* gate as is used in the designed circuit of this work. Therefore, an adaptation of eq 7 to emulate an internal gate response function model was implemented in this work.

All equations treated until now are mechanistic models derived from the Hill equation that describe the steady-state response function of each gate. However, to describe mechanistically, the response function *between* steady states would require many kinetic parameters that are difficult to empirically measure. Instead, a simplified model is shown in eq 8 that bundles all of these parameters into τ_ON_ and τ_OFF_, which captures the different mechanisms underlying the return to the steady state when the gate output is higher or lower than the steady-state value. This means that τ_ON_ is the response to go to a steady state that is higher than the current output and τ_OFF_ is the one to go to a steady state that is lower. These two parameters are the phenomenological measured observations of multiple kinetic rates like the production, degradation, or dispersion rates present in a cell.^45^ To describe the dynamic behavior of the internal gates, a set of ODEs for each genetic gate is needed to describe the time scale by which a gate turns *ON* or *OFF*, using a simplified model that uses only two parameters (τ_*y*_^ON^ and τ_*y*_^OFF^) as shown in the following equation
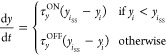
8

Equation 8 describes the dynamical response of each gate using the τ_ON_ and τ_OFF_ parameters, obtained in the Results section, where *y*_*i*_SS__ is the RNAP flux of gate *i* at steady state (eq 1), *y*_*i*_ is the current RNAP flux of gate *i*, and τ_*y*_^ON^ and τ_*y*_^OFF^ are the bundled kinetic parameters that capture the response time to go to a steady state that is higher than the current output (τ_*y*_^ON^) or lower (τ_*y*_^OFF^)^45,56^ and were set to an average value^45^ (see the Supporting Information). Finally, to calculate the RPU output of the promoter controlling YFP expression we use eq 9
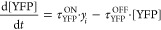
9where τ_YFP_^ON^ and τYFP^OFF^ are bundled kinetic parameters that capture the response time to go to a steady state that is higher than the current output (τ_YFP_^ON^) or lower (τYFP^OFF^),^45,56^*y*_*i*_ is the nonadditive input RNAP flux of the previous internal gate, and [YFP] is the fluorescence output measuring YFP expression.

The values for the parameters of the first noninformed model predictions were taken from the literature^45^ or used averages for when there were no values for certain gates (i.e., for sensor gates used as internal gates). However, after the recharacterization of the parts, these parameter values were changed to use the parametrization values (see the Results section). The resulting complete model is then analyzed using the Runge–Kutta–Fehlberg (4,5) method^36^ implemented in iBioSim,^35^ using the Synthetic Biology Open Language^57^ to describe the design. The model and parameter values can be seen in Supporting Information (Tables S1 and S2).

### Parametrization Methods

#### Hill Function Characterization Algorithm

For the Hill function parametrization method, a normalized least-squares method using the nonlinear *Least-Squares Minimization and Curve-Fitting* (lmfit) Python package^49^ was used, and random initial parameter estimations following the GAMES workflow.^58^

#### ON/OFF and OFF/ON Characterization Algorithm

The lmfit Python package, which is based on the Levenberg–Marquardt minimization algorithm, was used to perform the fits and analyze the resulting parameter sets.^49^ The fits were performed by minimizing the sum of the square of the relative error between each measured data point and the same point in the corresponding model simulation. As with the Hill function characterization algorithm, a random initial parameter value search was implemented following the GAMES workflow,^58^ while simultaneously looking for the smallest chi-squared values for each fitting iteration. These scripts are listed in the Supporting Information documentation.

Using the estimated values of τ_YFP_^OFF^, shown in Table S3, and using both eqs 4 and 5, the first fitting iteration was used to obtain τ_YFP_^ON^, τ_*x*_^ON^, and *x*_ss_ parameter estimate values using the *ON-to-OFF* characterization experiment results. Using the parameter estimation method proposed in ref (58) and the fixed values of τ_YFP_^OFF^ obtained previously, the model was fitted to the experimental results using a minimizing function. The parameter values estimated with this method are shown in Table S3.

Using the *ON-to-OFF* characterization experiments, and assuming that the influence of input sensor promoter flux is zero, then fitting eq 5 to the gradient of the fluorescence loss over time produces estimates of τ_YFP_^OFF^ parameter values.

### Plasmid Preparation

#### Circuit Plasmid (SZT61)

pAN3944 containing the AraC gate sequence, pAJM.477 containing the Lux_R sequence, and pAN4023 containing the Lux_R relevant ribozyme (RiboJ), RBS (BBa_B0064_rbs), and terminator (L3S2P21 terminator) were a gift from Prof. Christopher Voigt (Addgene plasmids #74702, #108526 and #74701, accordingly). The Lux_R sequence from pAJM.477 was amplified using the *polymerase chain reaction* (PCR) and cloned into the relevant location at pAN4023 using a standard Gibson assembly reaction.^59^ Then, the entire Lux_R gate was amplified using PCR and cloned into pAN3944 by using a standard Gibson assembly reaction. The removal of other gates from pAN3944 was done using a one-step PCR.

#### Reporter Plasmid Containing YFP (SZT45)

Using a one-step PCR, the promoter of reporter plasmid pAN4023 was changed to pLux_Star.

#### Reporter Plasmid Containing the MS2 Lysis Protein (SZT65)

The lysis protein sequence was synthesized (genscript) and cloned into SZT45 using standard Gibson assembly reactions.^59^

#### Lux_R Gate Characterization Circuit Plasmid (On/Off) (SZT69)

AraC gate was removed from SZT61 and the LuxR promoter was changed from the pBAD to a constitutive promoter, J23105, using a reverse PCR.

#### AraC Gate Characterization Circuit Plasmid (On/Off) (SZT70)

LuxR gate was removed from SZT61 using a reverse PCR.

#### AraC Gate Characterization Reporter Plasmid (On/Off) (SZT71)

The pBAD promoter was amplified from pAN3944 and cloned into SZT45 instead of the pLuxStar promoter using the standard Gibson assembly reaction.

### Circuit Induction and Measurements

The genetic circuit plasmid (SZT61) and the relevant reporter plasmid (SZT45 or SZT65) were cotransformed into chemically competent NEB 10-beta (New England Biolabs, MA, C3019) according to the manufacturer’s instructions. Following the transformation, the cells were plated on LB agar plates with 50 μg/mL kanamycin (Gold Biotechnology, K-120-5) and 50 μg/mL spectinomycin (Gold Biotechnology, MO, S-140-5). The plates were grown at 37 °C overnight, and single colonies were chosen and inoculated into 200 μL of M9 glucose with antibiotics in a deep 96-well plate (MasterBlock, 96 wells, PP, 2 mL). M9 glucose media were composed of M9 media salts (6.78 g/L Na2HPO4, 3 g/L KH2PO4, 1 g/L NH4Cl, 0.5 g/L NaCl), 0.34 g/L thiamine hydrochloride (Sigma-Aldrich, MO, T4625), 0.4% d-glucose (BDH), 0.2% casamino acids (Bacto), 2 mM MgSO4 (Fisher Chemicals), and 0.1 mM CaCl2. Antibiotic concentrations in M9 glucose medium were 50 μg/mL kanamycin and 50 μg/mL spectinomycin. The single colonies were grown at 37 °C overnight, 1000 rpm in Multitron Pro 2 shaker incubator. Following the incubation, the overnight cultures were diluted 178-fold by adding 15 μL of the culture into 185 μL of M9 glucose media and then 15 μL of that dilution into 185 μL of M9 glucose media with 50 μg/mL kanamycin and 50 μg/mL spectinomycin. For the different growth phases, the M9 media contained 2 μM *N*-Hexanoyl-l-homoserine lactone (HSL) (Sigma-Aldrich) and l-arabinose (Ara) (Sigma-Aldrich) was added at the relevant times to a final concentration of 5 mM. For the different concentrations of inducer assays, the M9 media contained the following inducer’s concentrations as shown in Table 1.

**Table 1:** Different Inducer Concentrations That Were Tested

condition	HSL (μM)	Ara (mM)
1:100	0.02	0.05
1:10	0.2	0.5
1:1	2	5
10:1	20	5

For the soil assays, the soil sample was split into two controls, sterile and nonsterile. The sterile soil was autoclaved, and the nonsterile soil was not. Then, the two controls were mixed with M9 media to a final concentration of 2% (W/V). The bacteria were grown according to the description above in M9 media and were diluted 178-fold into the soil media.

#### Fluorescence Measurements

The diluted culture was plated in a black 96-well plate with a clear bottom (655090, F-bottom, μclear, black, Greiner) and placed in a plate reader (Tekan SPARK plate reader) at 37 °C and 270 rpm. OD600 and fluorescence (excitation wavelength 485 nm and emission wavelength 535 nm) were measured every 10 min for at least 800 min. For the lysis protein assay, only the OD600 was measured. For the soil assay, only the fluorescence was measured. For the different temperature assays, the plate reader temperature was set to 30 and 42 °C.

#### ON/OFF Characterization Assays

For the AraC gate, AraC plasmid (SZT70) and the reporter plasmid (SZT71) were cotransformed into chemically competent NEB 10-beta (New England Biolabs, MA, C3019) according to the manufacturer’s instructions. For the LuxR gate, LuxR plasmid (SZT69) and the reporter plasmid (SZT45) were cotransformed into chemically competent NEB 10-beta (New England Biolabs, MA, C3019) according to the manufacturer’s instructions. Following the transformation, the cells were plated on LB agar plates with 50 μg/mL kanamycin (Gold Biotechnology, MO, K-120-5) and 50 μg/mL spectinomycin (Gold Biotechnology, MO, S-140-5). The plates were grown at 37 °C overnight and single colonies were chosen and inoculated into 200 μL of M9 glucose with antibiotics in a deep 96-well plate (MasterBlock, 96 wells, PP, 2 mL). M9 glucose media were composed of M9 media salts (6.78 g/L Na2HPO4, 3 g/L KH2PO4, 1 g/L NH4Cl, 0.5 g/L NaCl), 0.34 g/L thiamine hydrochloride (Sigma-Aldrich, MO, T4625), 0.4% d-glucose (BDH), 0.2% casamino acids (Bacto), 2 mM MgSO4 (Fisher Chemicals), and 0.1 mM CaCl_2_. Antibiotic concentrations in M9 glucose media were 50 μg/mL kanamycin and 50 μg/mL spectinomycin. The single colonies were grown at 37 °C overnight and 1000 rpm in a Multitron Pro 2 shaker incuabtor. Following the incubation, the overnight cultures were diluted 178-fold by adding 15 μL of the culture into 185 μL of M9 glucose media and then 15 μL of that dilution into 185 μL of M9 glucose media with 50 μg/mL kanamycin and 50 μg/mL spectinomycin. For the AraC gate, l-arabinose (Ara) (Sigma-Aldrich) was added at the relevant times to a final concentration of 5 mM. For the LuxR gate, HSL was added at the relevant times to a final concentration of 2 μM. The diluted culture was plated in a black 96-well plate with a clear bottom (655090, F-bottom, μclear, black, Greiner) and placed in a plate reader (Tekan SPARK plate reader) at 37 °C and 270 rpm. OD600 and fluorescence (excitation wavelength 485 nm and emission wavelength 535 nm) were measured every 10 min for 650 min. Following 650 min, the plates were centrifuged for 2 min at 4000 rpm and media were removed from each well. Then, fresh M9 media without any inducers were added to all the wells. The plates were then placed in the plate reader at 37 °C and 270 rpm. OD600 and fluorescence were measured every 10 min for an additional 650 min.

### Circuit Assay Analysis

For each sample, there were at least five biological repeats.

#### Fluorescence Graphs

The graphs represent the average values of these repeats. The fluorescence was normalized by subtracting the average blank value from the average fluorescence value and dividing the resulting fluorescence value by the average OD600 value for each time point.

#### Time for Fluorescence Detection Graphs

The normalized fluorescence values of the samples from *T* = 0 onward were compared to the normalized fluorescence values of the negative control (without induction). The time of fluorescence detection was determined as the time when the fluorescence values of the samples exceeded those of the negative control.

#### Fluorescence Fold Change Graphs

The maximum normalized fluorescence values of each sample were chosen. For the induction time variation assay, the *T* = 0 average was set as one and all the other samples were compared to it. For the inducer concentration variation assay, the 1:1 concentration average was set as one, and all the other samples were compared to it. For the inducer concentration variations at low and high temperatures, the 1:1 concentration average was set as one, and all the other samples were compared to it.

#### Time for Lysis Graphs

The normalized OD600 values of the samples from *T* = 0 onward were compared to the normalized OD600 values of the negative control (without induction). The time of lysis was determined as the time when the OD600 values of the samples decreased in comparison with the negative control.

#### Time for Rescue Graphs

The normalized OD600 values of the samples from the time of lysis were examined. The time of rescue was determined as the time when the OD600 values of the samples started to increase rather than decrease.

#### YFP (au) Production Rate Graphs

The sum of the normalized fluorescence values of each hour was calculated from the time of the fluorescence signal detection (see time for fluorescence detection graph explanation).

#### Doubling Time Graphs

The doubling time was determined as the time (min) it took for normalized OD600 values to double (from 0.2 to 0.4).

#### Confocal Images

The bacteria was cultivated and induced as described above for the lysis assay. At *T* = 6 h and *T* = 21 h, 200 μL from each sample was taken and centrifuged at 4000 rpm for 1 min. The cells were washed using 1 mL PBS and centrifuged. The pellet was resuspended with 50 μL of PBS. Then, live/dead staining was done according to the manufacturer’s instructions (L13152 LIVE/DEAD BacLight Bacterial Viability Kit, Molecular Probes, OR, USA), and the samples were subjected to confocal microscopy utilizing a LSM510 confocal microscope (Zeiss). The presented results are representative of three independent experiments.

## Supporting Information Available

Parameter values for default modeling and comparisons, parameter value estimations for delay circuit, doubling time assays, induction of bacteria at different growth phases, lysis protein circuit, iteratively fitted parameter value estimations to calculate τ_ON_, time for rescue of bacteria induced at different growth phases and with different inducers, vector maps for genetic circuit design, and gate characterization vectors. The sequence, data, modeling, and scripts used in this paper are also available at this GitHub repository: https://github.com/MyersResearchGroup/Shai-et-al-Supporting-Information (PDF)

## Footnotes

a https://synbiohub.programmingbiology.org/public/Eco1C1G1T1/Eco1C1G1T1_collection/1.
