# Supplementary material for: Investigating and Modeling the Factors That Affect Genetic Circuit Performance
Source: ACS Synth Biol. 2023 Nov 2;12(11):3189–204. doi: 10.1021/acssynbio.3c00151 (PMC10661042; doi:10.1021/acssynbio.3c00151)
Supplement: Supplementary file 1 — sb3c00151_si_001.pdf [file sb3c00151_si_001.pdf]

Supplementary Information: Investigating And Modeling the Factors that Effects the Performance of Genetic Circuits

Shai Zilberzwige-Tal[1,*], Pedro Fontanarrosa[2,⋆], Darya Bychenko[1], Yuval Dorfan[3,4], Ehud Gazit[1,5], and Chris J. Myers[2]

[1]The Shmunis School of Biomedicine and Cancer Research, Life Sciences Faculty, Tel Aviv University, 6997801, Israel
[2]Department of Electrical, Computer, and Energy Engineering, University of Colorado Boulder, 80309, USA
[3]Bio-engineering, Electrical engineering faculty, Holon Institute of Technology (HIT), 5810201, Israel
[4]Alagene Ltd., Innovation Center, Reichman University, 7670608, Israel
[5]The Department of Materials Science and Engineering, Engineering Faculty, Tel Aviv University, 6997801, Israel
[*]Corresponding author. shai.zil22@gmail.com
[⋆]Corresponding author. pfontanarrosa@gmail.com

September 25, 2023

Contents

List of Figures

S1 Doubling times . . . S5
S2 Induction of bacteria at different growth phases . . . S6
S3 Lysis protein circuit . . . S6
S4 Rescue times . . . S7
S5 Vectors map of the plasmids used for the delay circuit . . . S9
S6 Vectors map of the plasmids used for gates characterization . . . S10

List of Tables

S1 Input sensor gate parameters . . . S3
S2 Gate parameters . . . S3
S3 Estimation of Hill-function parameter values for different gates, obtained by fitting Equations 2 and 3 to part-characterization experiments using plasmids shown in Supplemental Figure S6 at different growth phases (EL: early-lag, LE: late-exponential), as explained in Section 2. The values are rounded to three significant digits. . . . S4
S4 Dynamic parameter values for different gates were estimated by fitting Equations 4 and 5 to *ON-to-OFF* and *OFF-to-ON* part-characterization experiments at different bacterial growth phases (EL: early-lag, LL: late-lag, EE: early-exponential, ME: middle-exponential, LE: late-exponential, S: stationary), as explained in Section 2 of this work. The experiments used the gate plasmids shown in Supplementary Figure S6. These characterizations were performed to determine if the dynamic behavior of the designed circuits varies with growth phases. The reported parameter values are rounded to three significant digits. . . S4

S5 Fitted parameter values for different iterative rounds. A stepwise iterative fitting process was performed to estimate parameter values for the model. The table shows the fitted parameter values for each round, with a different parameter fixed (first column) using the average of the previous round. The process continued until only one parameter (τ_y^{ON}) remained as a free variable. S7

Parameter Values Estimations

The parameters values used in this work's model predictions are described in this section. The values for the parameters of the first non-informed model predictions were taken from literature [1], stored in SynBioHub. or used averages for when there were no values for certain gates (i.e. for sensor gates used as internal gates). The parameter values used for the rest of the model predictions were obtained from a fitting algorithm using the lmfit Python package [2]. The data sources and scripts are available in the GitHub repository: `https://github.com/MyersResearchGroup/Shai-et-al-Supporting-Information`.

Non-informed default parameter values

Response function parameters

Table S1: Input sensor gate parameter values. This table summarizes the parameters used to calculate input promoter activities in the model. Values obtained from Nielsen et al. [292016Nielsen et al.Nielsen, Der, Shin, Vaidyanathan, Paralanov, Strychalski, Ross, Densmore, and Voigt]

.

Gate	x_{min} [1]	x_{max} [1]
Tac	0.0034	2.8
Tet	0.0013	4.4
BAD	0.0082	2.5

Table S2: Gate parameter values. This table summarizes the parameters used to determine promoter activities of the circuit. Values obtained from Nielsen et al. [292016Nielsen et al.Nielsen, Der, Shin, Vaidyanathan, Paralanov, Strychalski, Ross, Densmore, and Voigt].

Gate	y_{min} [1]	y_{max} [1]	κ [1]	n [2]
AmtR	0.06	3.8	0.07	1.6
BetI	0.07	3.8	0.41	2.4
BM3R1	0.01	0.8	0.26	3.4
HlyIIR	0.07	2.5	0.19	2.6
PhlF	0.02	4.1	0.13	3.9
SrpR	0.007	2.1	0.1	2.8

Gate dynamics

We used generic values for τ_{ON} and τ_{OFF} parameters obtained from [1]. The values used in this work are $\tau_{ON} = 1.7\ auRPU^{-1}hr^{-1}$ and $\tau_{OFF} = 4.044444444\ auRPU^{-1}hr^{-1}$.

Parameter Value Estimations

Hill Function Parameter Value Estimations

Dynamic Parameters (τ_{ON} and τ_{OFF}) Value Estimations

Doubling time

Doubling time across different assays.

Table S3: Estimation of Hill-function parameter values for different gates, obtained by fitting Equations 2 and 3 to part-characterization experiments using plasmids shown in Supplemental Figure S6 at different growth phases (EL: early-lag, LE: late-exponential), as explained in Section 2. The values are rounded to three significant digits.

	AraC			
Growth-phase	y_{max}	y_{min}	κ	n
EL	1730	342	4.16×10^{-2}	2.60
LE	1150	366	6.68×10^{-2}	2.89
	LuxR			
EL	4310	223	2.33	8.40×10^{-1}
LE	3230	308	5.52×10^{-1}	8.08×10^{-1}

Table S4: Dynamic parameter values for different gates were estimated by fitting Equations 4 and 5 to *ON-to-OFF* and *OFF-to-ON* part-characterization experiments at different bacterial growth phases (EL: early-lag, LL: late-lag, EE: early-exponential, ME: middle-exponential, LE: late-exponential, S: stationary), as explained in Section 2 of this work. The experiments used the gate plasmids shown in Supplementary Figure S6. These characterizations were performed to determine if the dynamic behavior of the designed circuits varies with growth phases. The reported parameter values are rounded to three significant digits.

	AraC			
Growth-phase	τ_x^{ON}	τ_{YFP}^{ON}	τ_{YFP}^{OFF}	x_{ss}
EL	0.0822	0.126	0.0893	976
LL	0.169	0.109	0.0933	1000
EE	0.117	0.13	0.114	1000
ME	0.111	0.112	0.111	999
LE	0.275	0.0749	0.096	998
S	0.208	0.0538	0.105	1000
	LuxR			
EL	0.143	0.335	0.166	995
LL	0.195	1.3	0.221	211
EE	0.11	0.274	0.11	938
ME	0.173	0.234	0.173	977
LE	0.0729	0.173	0.0726	1000

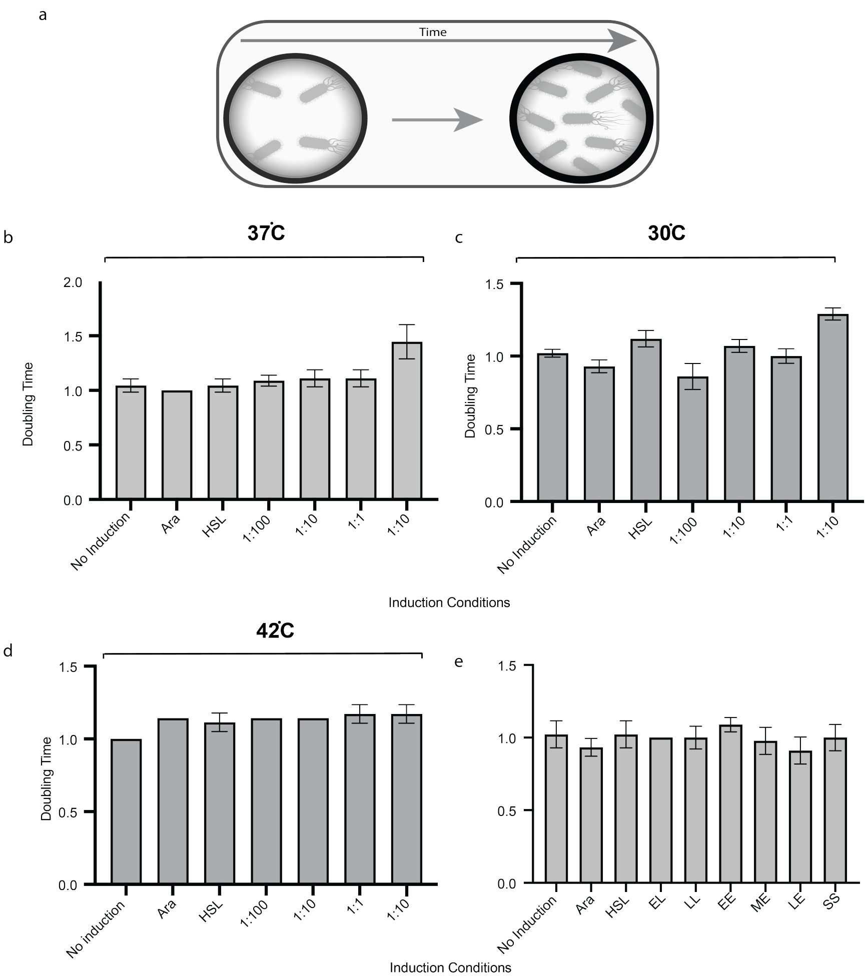

Figure S1: a. A scheme of the doubling time assay, see methods. b-d. Comparison between the averaged doubling times of the bacteria induced with a range of inducers' concentrations and at different temperatures: b. 37°C, c. 30°C and d. 42°C. ****$P < 0.001$, student t-test. e. Comparison between the averaged doubling times of the bacteria induced at different growth phases.

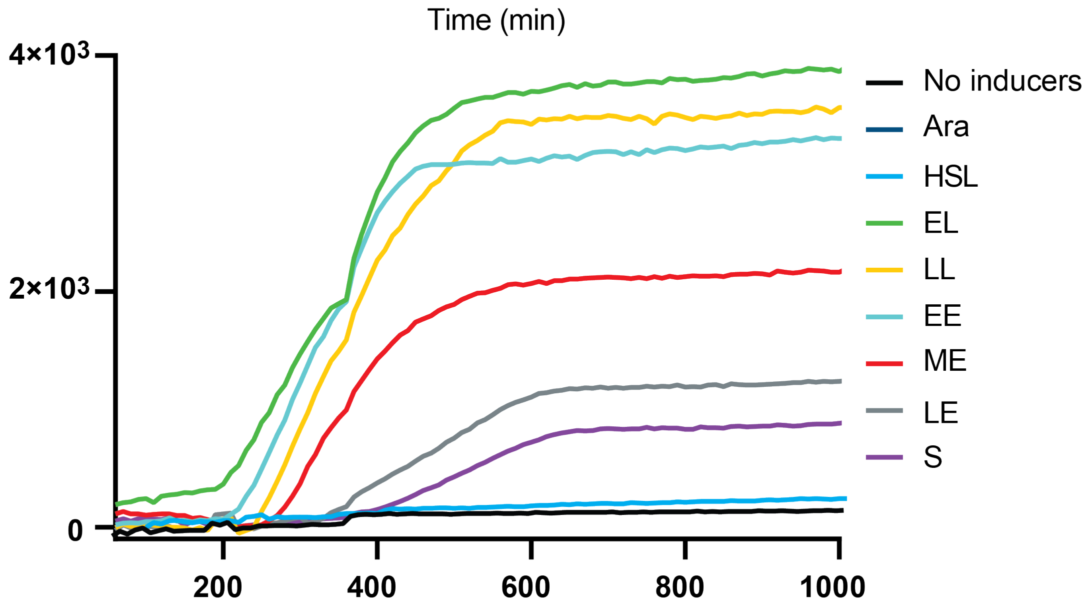

Figure S2: Induction of bacteria at different growth phases. Fluorescence signal over time of bacteria induction at different growth phases without normalization to the time of signal detection.

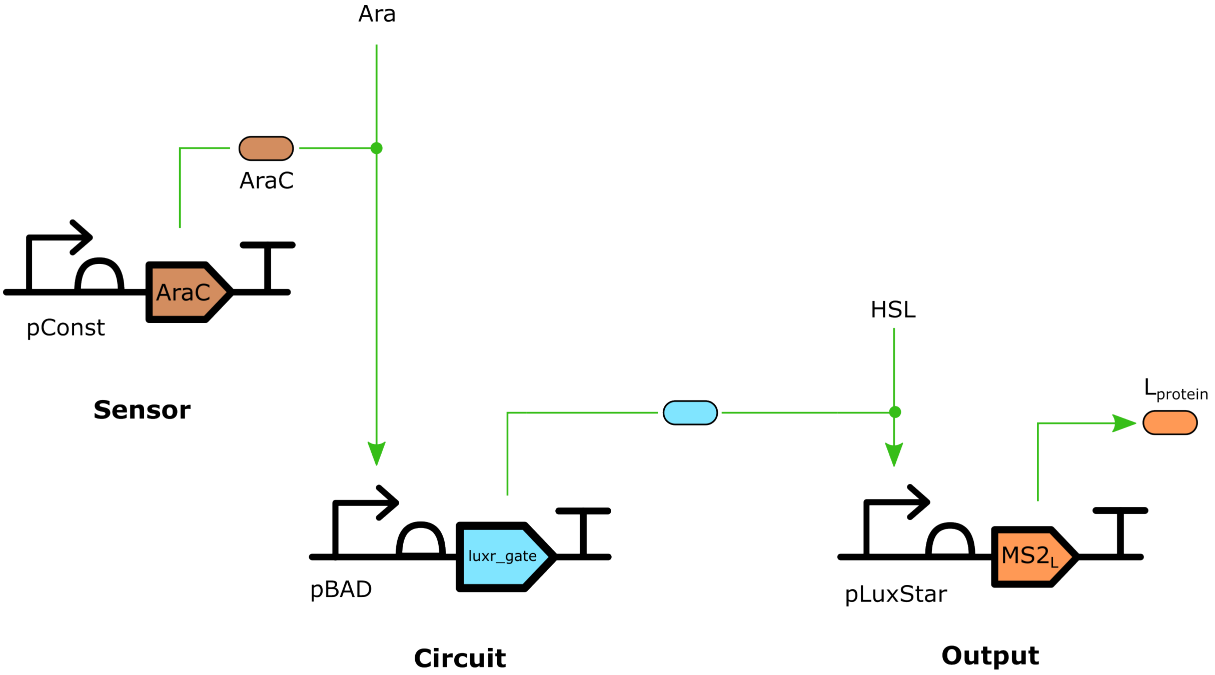

Figure S3: Lysis protein circuit. This circuit is designed to work the same as the delay circuit, but having a lysis protein as an output. This was used to study how changing the output of a circuit might affect the delay of the circuit.

Table S5: Fitted parameter values for different iterative rounds. A stepwise iterative fitting process was performed to estimate parameter values for the model. The table shows the fitted parameter values for each round, with a different parameter fixed (first column) using the average of the previous round. The process continued until only one parameter (τ_y^{ON}) remained as a free variable.

		t=0	t=180	t=210	t=240
**Fixing** x_{ss}	x_{ss}	1000	1000	1000	1000
	τ_{YFP}^{OFF}	0.0929173	0.0929173	0.0929173	0.0929173
	τ_x^{ON}	0.0454804	0.14426456	0.10057527	0.18635827
	τ_{YFP}^{ON}	0.17424227	0.10848398	0.10720921	0.09392156
**Fixing:** x_{ss}, τ_{YFP}^{OFF}	x_{ss}	1000	1000	1000	1000
	τ_{YFP}^{OFF}	0.0929173	0.0929173	0.0929173	0.0929173
	τ_x^{ON}	0.02553312	0.17034814	0.10057527	0.13492742
	τ_{YFP}^{ON}	0.17424227	0.10848398	0.10720921	0.09392156
**Fixing:** x_{ss}, τ_{YFP}^{OFF}, τ_x^{ON}	x_{ss}	1000	1000	1000	1000
	τ_{YFP}^{OFF}	0.0929173	0.0929173	0.0929173	0.0929173
	τ_x^{ON}	0.1008304	0.1008304	0.1008304	0.1008304
	τ_{YFP}^{ON}	0.12111371	0.11351539	0.11132977	0.09662176

Induction of bacteria at different growth phases

Lysis Protein Circuit Design

Lysis rescue

Time for rescue of bacteria induced at different growth phases and with different inducers' concentrations.

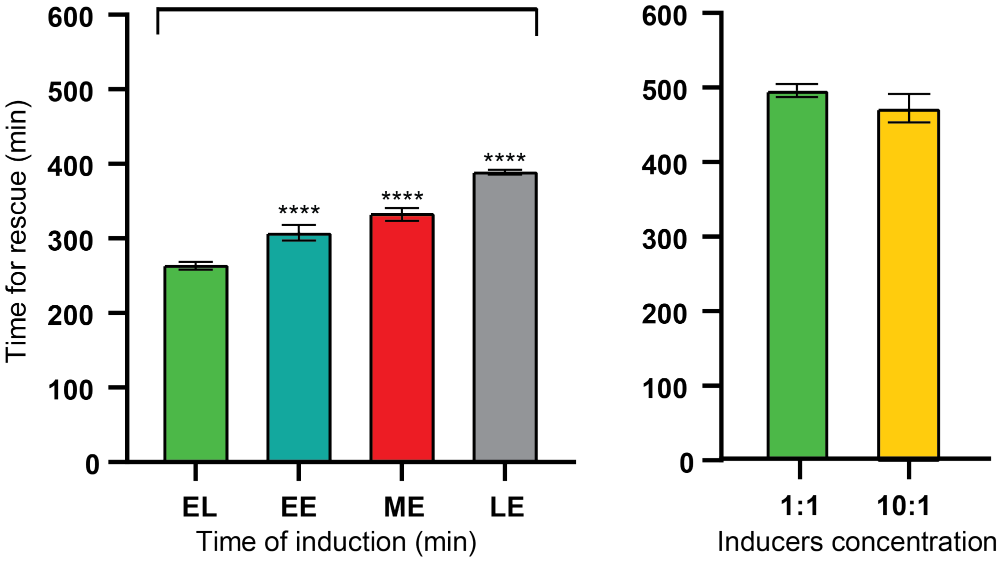

Figure S4: Time for rescue of bacteria induced at different growth phases and with different inducers' concentrations. ****$p < 0.0001$ student t-test.

[1] In units of RPU [3].

[2] Dimensionless.

Vectors map

The different vectors that were used.

Delay circuit vectors

Gates characterization vectors

References

[1] Jonghyeon Shin, Shuyi Zhang, Bryan S Der, Alec AK Nielsen, and Christopher A Voigt. Programming *Escherichia coli* to function as a digital display. *Molecular Systems Biology*, 16(3):e9401, March 2020.

[2] Matt Newville, Renee Otten, Andrew Nelson, Antonino Ingargiola, Till Stensitzki, Dan Allan, Austin Fox, Faustin Carter, Michał, Ray Osborn, Dima Pustakhod, lneuhaus, Sebastian Weigand, Glenn, Christoph Deil, Mark, Allan L. R. Hansen, Gustavo Pasquevich, Leon Foks, Nicholas Zobrist, Oliver Frost, Alexandre Beelen, Stuermer, azelcer, Andrew Hannum, Anthony Polloreno, Jens Hedegaard Nielsen, Shane Caldwell, Anthony Almarza, and Arun Persaud. Lmfit/lmfit-py: 1.0.3. Zenodo, October 2021.

[3] Jason R Kelly, Adam J Rubin, Joseph H Davis, Caroline M Ajo-Franklin, John Cumbers, Michael J Czar, Kim de Mora, Aaron L Glieberman, Dileep D Monie, and Drew Endy. Measuring the activity of biobrick promoters using an in vivo reference standard. *Journal of biological engineering*, 3(1):1–13, 2009.

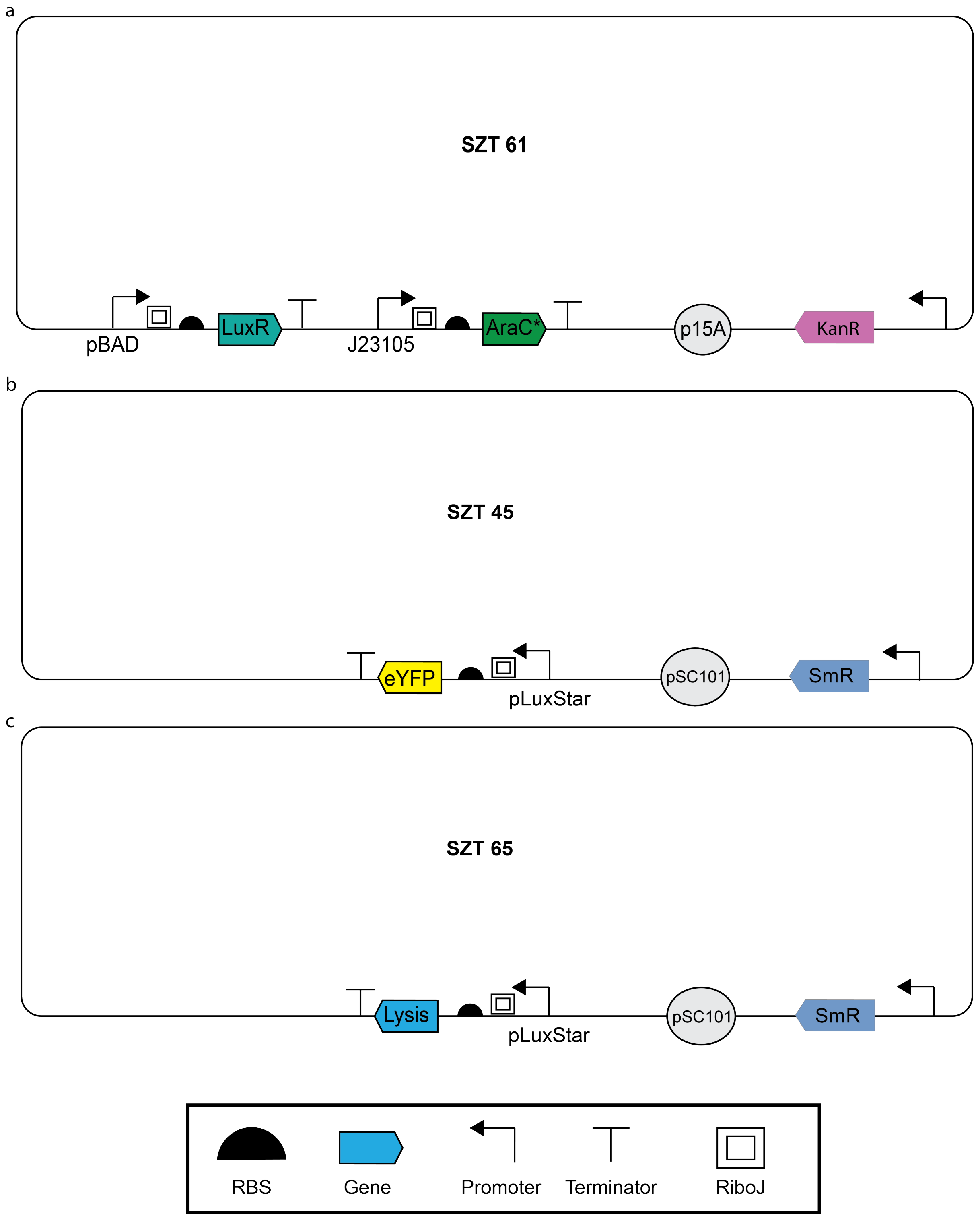

Figure S5: Vector maps of a. delay circuit, b. reporter plasmid encoding YFP, c. reporter plasmid encoding lysis protein.

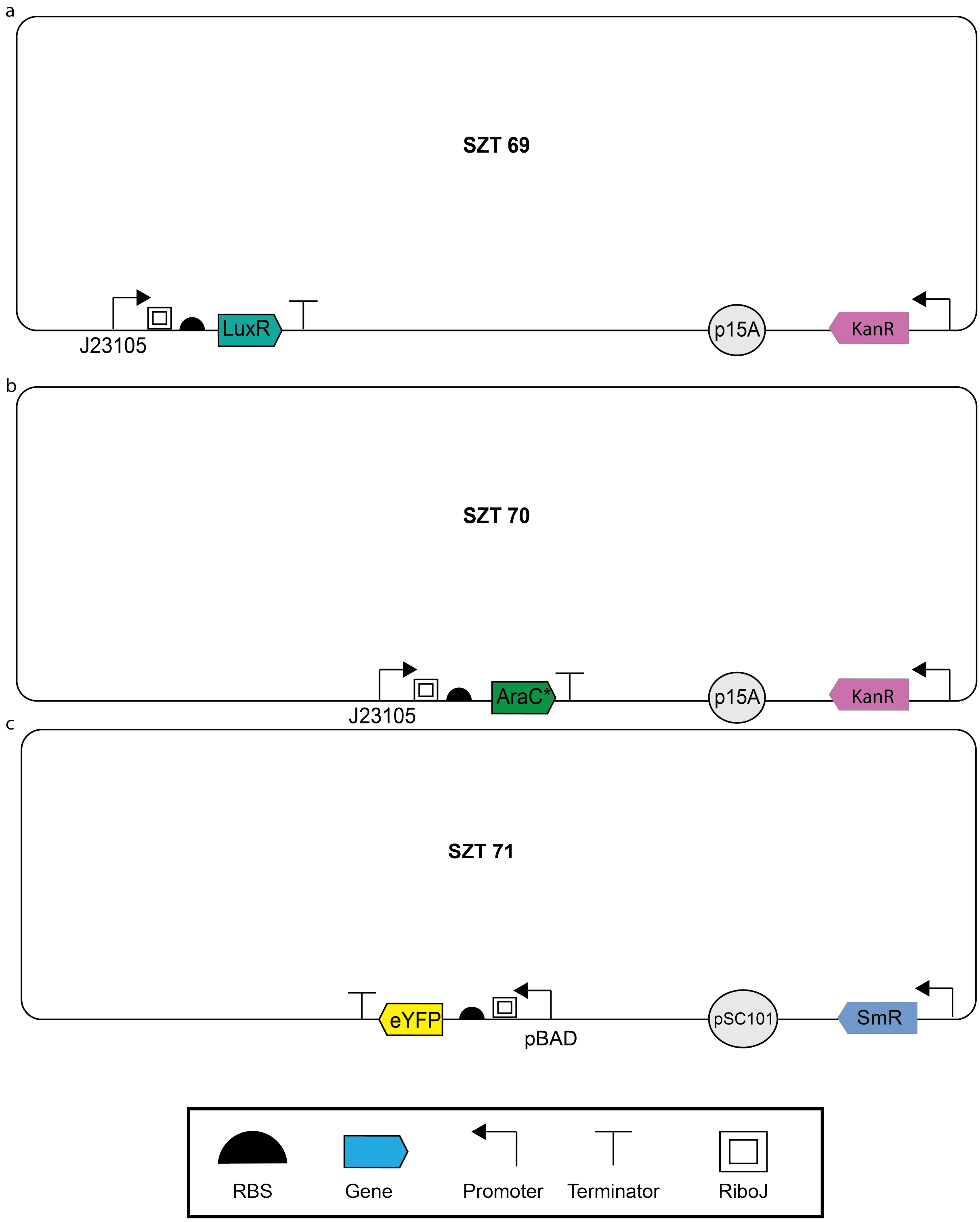

Figure S6: Vector maps of a. LuxR gate characterization, b. AraC gate characterization, c. reporter plasmid for AraC gate characterization.
